# Sustainable Hierarchical Networking for Extreme-Edge AI-Based Wildlife Monitoring in Resource-Constrained Environments

**DOI:** 10.3390/s26185942

**Published:** 2026-09-19

**Authors:** Md Auhidur Rahman, Ervisa Marku, Sunil Mathew, Davide Adami, Michele Pagano, Stefano Giordano

**Affiliations:** 1 Department of Information Engineering, University of Pisa, Via G. Caruso 16, 56122 Pisa, Italy; ervisa.marku@ing.unipi.it (E.M.); sunil.mathew@ing.unipi.it (S.M.); michele.pagano@unipi.it (M.P.); stefano.giordano@unipi.it (S.G.); 2 Software Engineering Program, Institute of Information Technology, Noakhali Science and Technology University, Noakhali 3814, Chattogram, Bangladesh; 3 Department of Information Engineering, CNIT, University of Pisa, 56122 Pisa, Italy; davide.adami@iet.unipi.it

**Keywords:** IEEE 802.11ah, networking for AI, YOLO, extreme edge, wildlife monitoring, sustainable networking for AI, sustainable approaches for AI, model quantization, edge deployments

## Abstract

Deploying artificial intelligence (AI) for wildlife monitoring in remote environments remains challenging due to limited energy, computational resources, and unreliable communication. Conventional edge AI systems rely on continuous sensing, high-power communication, and full-precision models, resulting in excessive energy consumption. This paper presents a green-energy-based hierarchical network architecture for sustainable wildlife monitoring at the extreme edge. The framework distributes sensing, communication, and AI inference across edge, far-edge, and extreme-edge layers. A low-power IEEE 802.11ah network with on-demand channel allocation reduces transmission overhead, while passive infrared (PIR)-based event-driven activation replaces continuous camera operation. Model quantization (FP32, FP16, INT8) and distributed split inference improve computational efficiency, and tracking-assisted communication eliminates redundant transmissions. Experimental results demonstrate significant sustainability improvements. PIR-based operation reduces daily energy consumption by 95.11% compared with continuous operation. Tracking-assisted communication reduces both bandwidth requirements and energy consumption by more than 99% (p<0.001). Token-based channel allocation provides 39.4% energy savings compared with Target Wake Time (TWT)-based scheduling (sensitivity range: 29.2–45.1%). INT8 quantization delivers 4.7× higher throughput, 61% lower memory usage, and 39% lower power consumption, with a 2.6% mean average precision (mAP) reduction. Field validation confirms the INT8 model’s effectiveness with an overall mAP of 82.89% (86.40% daytime, 79.37% nighttime) and precision above 96%. Combined with PIR activation, the INT8 system achieves 63.76 Wh/day—a 95.17% reduction versus FP32 continuous operation. The integration of renewable energy harvesting, efficient sensing, lightweight AI, and communication optimization enables scalable, autonomous, and sustainable wildlife monitoring in resource-constrained environments.

## 1. Introduction

Wildlife monitoring plays a critical role in biodiversity conservation, ecosystem management, and the mitigation of human–wildlife conflicts [1]. Traditional monitoring approaches, such as manual surveys, camera trapping, and GPS-based tracking, are often labor-intensive, costly, and difficult to scale [2]. Moreover, these methods generally lack real-time responsiveness and are difficult to deploy in remote or hostile environments where continuous human intervention is impractical. Consequently, there is an increasing demand for intelligent and automated monitoring systems capable of operating autonomously under resource-constrained conditions [3]. Recent advances in AI, particularly deep learning-based object detection, have enabled accurate and real-time wildlife monitoring [4,5]. AI-driven approaches significantly outperform conventional methods in terms of detection accuracy, adaptability, and scalability [5]. However, deploying such systems in extreme-edge environments introduces several challenges, including limited energy availability, intermittent connectivity, constrained computational resources, and sustainability concerns associated with power consumption and carbon emissions [6,7]. These challenges necessitate the development of energy-aware and distributed edge AI architectures that support environmentally sustainable operation [8]. To address these issues, hierarchical network architectures for AI have emerged as an effective solution for extreme-edge deployments [9,10,11]. In such architectures, sensing, processing, and decision-making tasks are distributed across multiple network layers, thereby reducing dependence on centralized cloud infrastructures and minimizing communication overhead [12,13]. Reducing unnecessary communication and computation not only improves energy efficiency but also helps reduce carbon emissions associated with network operation and cloud-based processing [13,14]. Sustainable connectivity is a key requirement in hostile and remote environments [7,8]. For mid-haul communication, point-to-point (PtP) sub-6 GHz 550E wireless links are employed to provide reliable and energy-efficient data transmission between distributed edge infrastructures [15]. For fronthaul communication between extreme edge nodes and the access point (AP), IEEE 802.11ah (Wi-Fi HaLow) is used because of its long-range and low-power communication capabilities [16,17]. Although IEEE 802.11ah incorporates energy-efficient MAC mechanisms, such as Target Wake Time (TWT), these mechanisms are primarily designed for periodic and predictable traffic patterns, in which stations communicate at pre-negotiated wake-up intervals [18,19]. In the considered wildlife monitoring scenario, however, traffic generation is event-driven and bursty, because it depends on real-time object detections, making communication highly asynchronous and unpredictable.

To address this limitation, a token-based channel allocation scheme is adopted instead of conventional TWT scheduling. Upon detecting a wildlife event, an extreme edge node immediately requests a transmission token from the AP, which dynamically grants channel access for the delivery of time-series Internet of Things (IoT) data. This event-synchronized communication mechanism reduces contention, avoids delays associated with fixed wake-up schedules, and improves responsiveness under bursty traffic conditions. The design is further motivated by studies showing that contention-aware and traffic-aware MAC mechanisms can significantly improve channel utilization and reduce communication overhead in dense IEEE 802.11ah IoT networks [20,21]. For backhaul connectivity in remote environments, low-Earth-orbit (LEO) satellite communication is considered a viable solution for connecting the edge infrastructure to cloud services and remote monitoring centers [22].

Unlike conventional always-on communication systems, the proposed architecture enables network nodes to remain in low-power states and transmit data only when required [7]. This design significantly reduces energy consumption, extends the operational lifetime of deployed devices, and minimizes the carbon footprint of the monitoring system. Another important aspect of sustainable network design is the development of extreme-edge AI processing nodes capable of integrating multiple IoT-based sensing devices for local decision-making [23]. To achieve sustainability, both hardware- and software-level optimization techniques are required to minimize overall power consumption and associated environmental impact.

At the hardware level, sustainability is further enhanced through the integration of passive infrared (PIR) sensors for event-driven processing [24]. The PIR sensor acts as a trigger mechanism that activates the edge computing unit, based on the NVIDIA Jetson Orin Nano, only when motion is detected within the monitoring zone. This eliminates the need for continuous camera operation and unnecessary AI processing, thereby reducing computational load, minimizing redundant data generation, and lowering energy usage [24,25]. As a result, communication occurs only when meaningful events are detected, further improving sustainability and reducing carbon emissions.

In addition to hardware-assisted optimization, software-level sustainability is achieved through the integration of object detection and tracking [26,27]. Conventional detection-only systems often generate redundant frames for the same animal across consecutive video frames, leading to unnecessary processing, repeated data transmission, increased bandwidth usage, and  higher power consumption. Moreover, frame-based detection alone may result in inaccurate or duplicated animal counting. To address this issue, the proposed system integrates detection with tracking by assigning unique track IDs to detected animals [27]. This enables event-driven monitoring, where only meaningful and non-redundant information is transmitted to edge servers. Consequently, the system significantly reduces communication overhead, bandwidth utilization, and energy consumption while improving animal-counting accuracy and overall monitoring efficiency. Therefore, detection with tracking plays an important role in achieving sustainable AI-based wildlife monitoring. Furthermore, computational efficiency is achieved through model-level optimization techniques. In this work, model quantization methods using FP16 and INT8 precision are employed to significantly reduce memory usage and computational complexity compared with conventional FP32 models [28,29]. Quantization enables faster inference and lower power consumption on edge devices, making it highly suitable for real-time wildlife monitoring applications. Reduced computational demand directly contributes to lower energy consumption and decreased carbon emissions during long-term operation [28]. Compared with model pruning, which may introduce irregular sparsity and hardware inefficiencies, quantization provides a more practical and hardware-friendly optimization strategy [30]. In addition, a split inference strategy is adopted by decomposing the trained object detection model (best.pt) into two separate components: backbone.pt and neck_head.pt. This architectural separation allows feature extraction to be performed at the extreme edge [31], while higher-level processing can be selectively executed on more capable edge or intermediate nodes. Such partitioning reduces the computational burden on individual devices, optimizes resource utilization across the hierarchical network, and further contributes to energy-efficient and sustainable AI deployment.

By combining hierarchical networking, IEEE 802.11ah-based on-demand communication, PIR-triggered event-driven sensing, detection with tracking, quantized AI models, and split inference techniques, the proposed system establishes a sustainable and energy-efficient framework for wildlife monitoring in hostile environments. This integrated approach effectively reduces power consumption, bandwidth utilization, and carbon emissions across communication, sensing, and computation layers while ensuring reliable, scalable, and real-time operation in challenging deployment scenarios. The main contributions of this work are summarized as follows: 1. Design and development of a sustainable hierarchical “Network for AI” architecture for hostile and remote environments, enabling distributed sensing, edge intelligence, and scalable wildlife monitoring under resource-constrained conditions. 2. Reduction of communication-related power consumption through the integration of PIR-triggered event-driven activation combined with AI-based object detection and tracking. This approach minimizes unnecessary sensing, processing, and data transmission by enabling communication only when meaningful events are detected. 3. Reduction of computational power consumption on extreme-edge processing devices through model quantization techniques using FP16 and INT8 precision. These techniques significantly reduce memory usage and computational complexity while maintaining efficient real-time AI inference on embedded edge hardware such as the NVIDIA Jetson Orin Nano. 4. Development of a remote monitoring and management framework for extreme-edge AI nodes, enabling real-time health monitoring, failure detection, and remote maintenance of deployed systems. The proposed framework utilizes Docker container-based deployment, allowing remote model updates, processing reconfiguration, and rapid deployment of new AI services without physical access to the nodes. In addition, the system supports remote identification of malfunctioning or inactive nodes, improving reliability, maintainability, and scalability in hostile deployment environments.

The remainder of this paper is organized as follows. Section 2 reviews related work and provides the background for the proposed framework. Section 3 describes the proposed methodology, including hardware integration and design of the hierarchical network architecture for extreme-edge AI. Section 4 describes how the proposed framework supports sustainability through energy-efficient sensing, communication, and computation based on the joint optimization of hardware and software components. Section 5 provides a load analysis of the extreme-edge processing nodes and AP, followed by the sizing of the solar photovoltaic system and battery capacity for long-term autonomous operation. Section 6 explains the operational workflow of the proposed framework. Section 7 presents the experimental results and discusses the performance of the proposed system in terms of detection accuracy, communication efficiency, computational performance, energy consumption, and calculated system autonomy. Section 8 provides statistical analyses and visualizations of wildlife detection, sensing, communication, and other system-level performance metrics. Finally, Section 9 concludes the paper and outlines directions for future research.

## 2. Related Works

### 2.1. AI-Based Wildlife Monitoring

Wildlife monitoring has increasingly adopted AI-based approaches to improve automation, detection accuracy, and scalability. Deep learning-based object detection models such as YOLO have demonstrated significant success in detecting and classifying wildlife species in real-time environments [3,5]. AI-driven monitoring systems provide advantages over conventional approaches such as manual surveys and camera trapping by enabling autonomous operation and faster data analysis [4]. Recent studies have also explored the use of embedded AI systems for wildlife monitoring in remote environments. However, many existing systems still rely heavily on centralized cloud infrastructures and continuous data transmission, leading to increased bandwidth usage and energy consumption.

### 2.2. Edge and Hierarchical AI Architectures

To address latency and bandwidth limitations of cloud-centric systems, edge computing and hierarchical edge–fog–cloud architectures have emerged as promising solutions [6,8]. Distributed architectures enable sensing, processing, and  decision-making tasks to be performed closer to data sources, reducing communication overhead and improving response time. Several studies have investigated hierarchical computing frameworks for IoT and AI applications [9,10,11]. These architectures support distributed intelligence and resource optimization across multiple network layers. Nevertheless, existing approaches often prioritize performance and scalability while giving limited consideration to sustainability, carbon footprint reduction, and energy-aware wildlife monitoring deployments in hostile environments (see Table 1).

### 2.3. Sustainable Communication and Event-Driven Systems

Energy-efficient communication has become an important research topic for extreme-edge and IoT systems. IEEE 802.11ah (Wi-Fi HaLow) and low-power wireless communication technologies have been proposed to support long-range and energy-aware communication in constrained environments [16]. Similarly, event-driven sensing mechanisms using sensors such as PIR detectors have been explored to reduce unnecessary sensing and processing activities [24]. These approaches enable edge nodes to remain in low-power states and activate processing only when meaningful events occur. Although prior works demonstrate improved energy efficiency, the integration of event-driven sensing with AI-based wildlife detection, tracking, and sustainable communication optimization remains limited.

### 2.4. Object Detection, Tracking, and Efficient AI Inference

Object detection and tracking techniques have been widely used in intelligent surveillance and monitoring systems. Multi-object tracking methods such as Deep SORT improve temporal consistency and reduce redundant detections by assigning persistent IDs to detected objects [26]. In wildlife monitoring applications, tracking can significantly reduce duplicate counting and redundant data transmission. In addition, model optimization techniques such as FP16 and INT8 quantization have been proposed to reduce computational complexity and power consumption for edge AI systems [28,29]. Split inference strategies have also emerged as efficient approaches for distributing AI workloads across edge and intermediate nodes [31]. However, limited research has jointly considered tracking-based communication reduction, quantized AI inference, and distributed split processing within a unified sustainable edge AI framework.

### 2.5. Remote Edge Management and Containerized Deployment

Remote management and orchestration of distributed edge nodes are critical for deployments in remote and hostile environments [32,33]. Containerization technologies such as Docker provide lightweight and scalable deployment mechanisms for AI services on edge devices [34]. Recent studies have highlighted the advantages of container-based deployment for remote software updates, service migration, resource isolation, and flexible AI application management in distributed IoT and edge computing systems [35,36]. Furthermore, orchestration and monitoring frameworks have been investigated to improve fault tolerance, scalability, and maintainability in distributed edge infrastructures [37]. However, many existing wildlife monitoring solutions lack integrated frameworks for remote node monitoring, failure detection, and adaptive deployment management across extreme-edge infrastructures.

### 2.6. Research Gap

Although significant progress has been made in AI-based wildlife monitoring, edge computing, and sustainable IoT communication, existing studies typically focus on isolated aspects of the problem [11,13]. Existing studies generally emphasize communication optimization, AI inference acceleration, or sensing efficiency in isolation, without considering a holistic sustainability-oriented framework for hostile extreme-edge environments. Few studies provide a unified architecture that jointly addresses sustainable networking, event-driven sensing, AI-based detection with tracking, quantized inference, split AI processing, and remote edge node management for wildlife monitoring applications [3,9]. Therefore, this work proposes a sustainable hierarchical Network for AI architecture that integrates communication, sensing, computation, and remote management optimizations to enable energy-efficient and scalable wildlife monitoring under constrained deployment conditions.

## 3. Hierarchical Network Design for AI

This section presents the hierarchical communication network design for AI systems. The network is organized into three layers: backhaul, midhaul, and fronthaul, which are mapped to the edge, far-edge (AP), and extreme-edge layers, respectively. This section outlines the overall system architecture, including the hardware configuration and software components supporting model training, inference, and deployment. The section also covers dataset preparation and model optimization through quantization, followed by a description of the communication framework with built-in fault tolerance for reliable operation in rural environments.

### 3.1. Communication Network Architecture for Sustainable Extreme Edge AI

**Figure 1:**
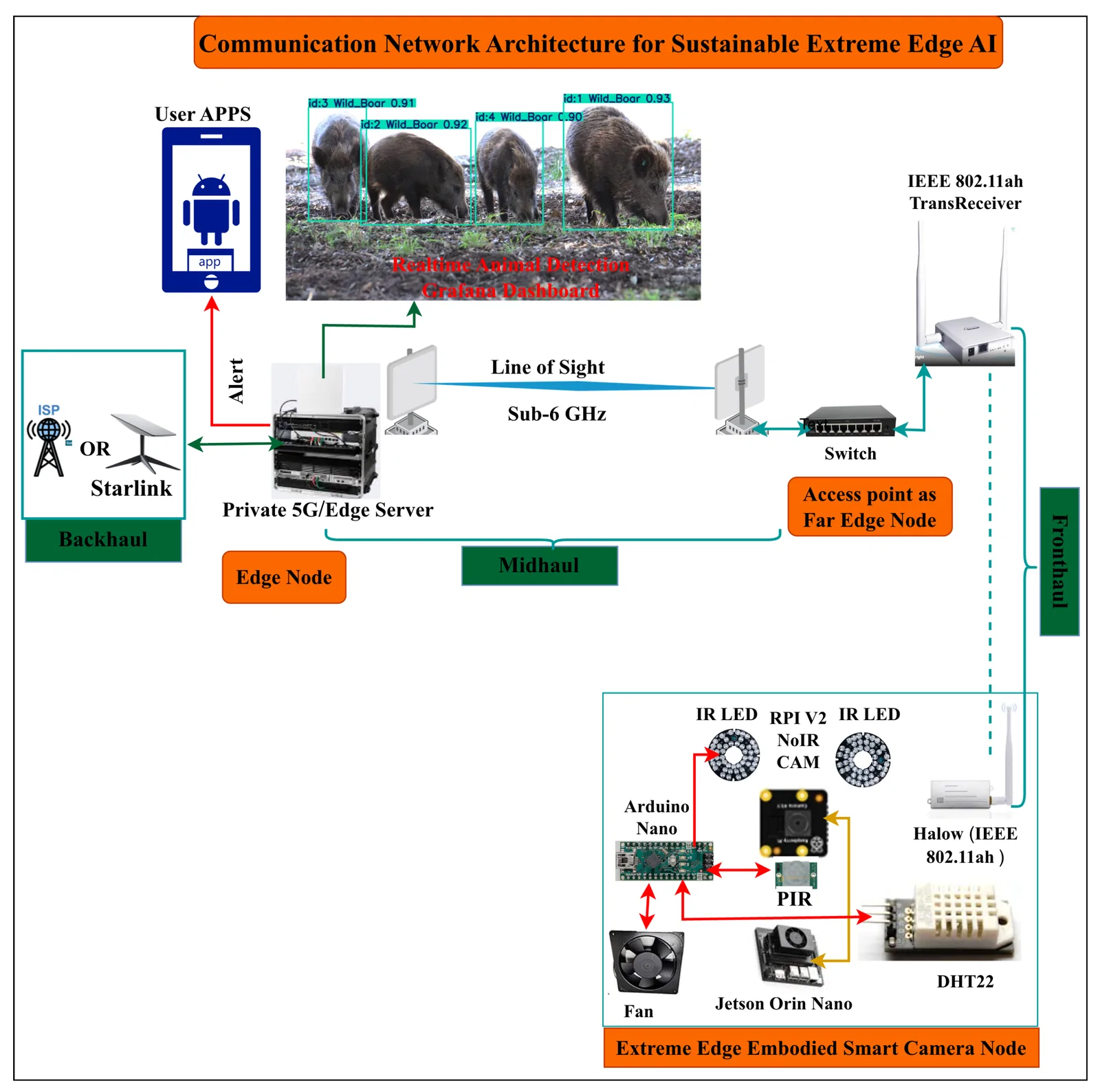
Communication Network Architecture.

The proposed green-energy-based sustainable extreme-edge AI Network is designed to enable real-time wildlife monitoring and rural safety in resource-constrained regions. The technical solution integrates energy-efficient sensors, low-power extreme-edge AI devices, lightweight inference models, and a robust multi-tiered communication network. The communication framework is divided into three layers, namely the backhaul, midhaul, and fronthaul in Figure 1. Each layer plays a distinct role in ensuring reliable and scalable data transmission under challenging rural conditions.

The backhaul layer serves as the primary connection between the deployment site and regional or cloud-based monitoring centers. In this layer, private 5G/edge server networks are proposed as the preferred option to guarantee high bandwidth, low latency, and secure connectivity. In situations where licensing or regulatory issues arise, alternative solutions such as Starlink LEO satellite links or conventional internet service providers (ISPs) can be adopted to establish reliable backhaul connections. These options ensure continuous data flow even in remote areas lacking stable terrestrial infrastructure.

The midhaul layer provides intermediate connectivity between community-level aggregation hubs and the backhaul network. The PTP 550E sub-6 GHz wireless links are proposed as a practical solution for this layer, particularly where direct line-of-sight (LoS) connections can be established for mid- and long-range applications. For the pilot deployment, a suitable example is the national park of San Rossore in Pisa, Italy, where two guard buildings are positioned with a clear line of sight. By utilizing PtP sub-6 GHz links between these buildings, the system can extend coverage deeper into the park, enabling monitoring in areas of high wildlife activity. This midhaul design ensures both reliability and flexibility, adapting to the geographical and infrastructural context of deployment.

The fronthaul layer connects the extreme-edge sensing devices to local aggregation nodes. This layer consists of APs equipped with Wi-Fi HaLow (IEEE 802.11ah) transceivers, which provide long-range, low-power connectivity suitable for rural deployments. Smart camera poles fitted with Wi-Fi HaLow modules act as edge nodes, communicating directly with fronthaul access points. The use of Wi-Fi HaLow ensures energy-efficient operation, robust penetration through vegetation and obstacles, and cost-effective deployment across wide monitoring zones. Together, the backhaul, midhaul, and fronthaul layers create a resilient and flexible communication architecture tailored for wildlife monitoring in rural and resource-constrained environments. This multi-tiered design ensures that the network can operate efficiently under varying infrastructure constraints while remaining scalable and adaptable for future extensions.

### 3.2. Connectivity Motivation

From the comparison of network communication technologies in Table 2, it is clear that different layers of the proposed architecture demand different connectivity solutions. For the fronthaul, Wi-Fi HaLow (IEEE 802.11ah) is chosen over LoRa/LoRaWAN because, while LoRa excels in ultra-low-power, small-payload applications, it cannot support the higher data rates required for edge inference results, multi-object tracking metadata, or occasional image/frame transfers. Wi-Fi HaLow, on the other hand, offers sub-GHz operation with long-range coverage up to 1 km, low energy consumption, and sufficient throughput, making it ideal for wildlife park deployments and extreme-edge smart camera poles.

For midhaul, PtP sub-6 GHz radio links provide reliable high-throughput connectivity (5–20 km line-of-sight), ensuring consistent data flow between distributed extreme-edge nodes and aggregation points such as ranger stations or guard posts. For the backhaul, satellite-based connectivity is required to ensure reliable communication in remote and rural regions where terrestrial networks may be unavailable. In this context, Starlink LEO provides a practical solution, offering global coverage and significantly lower latency compared to traditional geostationary satellite systems. This capability enables near real-time monitoring and data transfer from isolated conservation areas to central cloud platforms, thereby extending the reach of the proposed system to locations beyond the coverage of conventional 5G or fiber infrastructure.

Together, Wi-Fi HaLow, PtP links, and LEO satellite backhaul deliver a sustainable, scalable, and fault-tolerant communication layer tailored to the needs of rural edge AI deployments.

### 3.3. Hardware Setup

The hardware setup for the sustainable extreme-edge AI Network is designed around three primary layers: sensing, processing, and communication. Each layer is optimized to ensure low power consumption, robustness in rural environments, and long-term autonomous operation.

The sensing layer consists of multimodal devices shown in Figure 1 deployed at strategic locations such as roadsides, farm perimeters, forest edges, and village boundaries. These include camera modules, such as CSI cameras or thermal imaging units, which provide visual monitoring of wildlife activity. An example is the Raspberry PI Cam V2 (Sony Imx219 sensor), which supports daytime and nighttime monitoring. For nighttime operation, a 940 nm IR LED crown also needs to be integrated.

To reduce energy consumption, PIR motion sensors are integrated as event-driven triggers, activating cameras and edge processors only when motion is detected. Acoustic sensors further complement the visual systems by capturing animal calls or unusual sounds, thereby enhancing detection accuracy in low-visibility conditions such as nighttime, fog, or dense vegetation. In addition to wildlife detection, environmental context is captured using DHT22 sensors, which record temperature and humidity data. These environmental parameters are correlated with wildlife activity to better understand behavioral patterns and context-specific movements. Careful placement of these sensors ensures maximum coverage while minimizing redundancy and power consumption, ultimately creating a more energy-efficient and context-aware sensing infrastructure.

The processing layer is responsible for running lightweight AI inference models locally at the extreme edge. This layer uses low-power devices such as the NVIDIA Jetson Orin Nano (8 GB) or Orin boards. These platforms provide sufficient computational power for real-time inference while maintaining a low energy footprint. To support off-grid deployment, the devices are powered by solar energy systems coupled with battery backups, enabling continuous operation even during extended periods of limited sunlight. Enclosures for these units are designed to withstand harsh weather conditions and prevent damage from wildlife interactions, ensuring durability in field environments.

The communication layer provides connectivity between edge devices, community hubs, and backhaul infrastructure via Starlink LEO satellite. Multiple technologies are employed based on the deployment context: sub-6 GHz links for midhaul between edge server and AP, and IEEE 802.11ah (Wi-Fi HaLow) connections from the AP to extreme-edge smart camera nodes. A fault-tolerant design ensures fallback communication paths in case of link failures, maintaining uninterrupted data transfer. This resilience is critical for rural and remote deployments, where infrastructure reliability is often limited. Additionally, the edge server can trigger real-time alert notifications to user apps whenever detections occur locally. Together, the sensing, processing, and communication layers form an integrated hardware ecosystem that balances efficiency, resilience, and scalability, enabling sustainable wildlife monitoring and rural safety in resource-constrained environments.

## 4. Sustainability Approaches for the Network for AI

In this work, sustainability refers to the capability of the proposed wildlife monitoring system to maintain long-term autonomous operation in remote and resource-constrained environments while minimizing energy consumption, communication overhead, and maintenance requirements. Sustainability is achieved through the integration of renewable energy harvesting, event-driven sensing, energy-efficient AI inference, bandwidth-aware communication, and intelligent resource management. By reducing computational load, limiting unnecessary data transmissions, and extending battery autonomy, the proposed architecture lowers operational costs and environmental impact while ensuring reliable wildlife monitoring in hostile environments.

**Figure 2:**
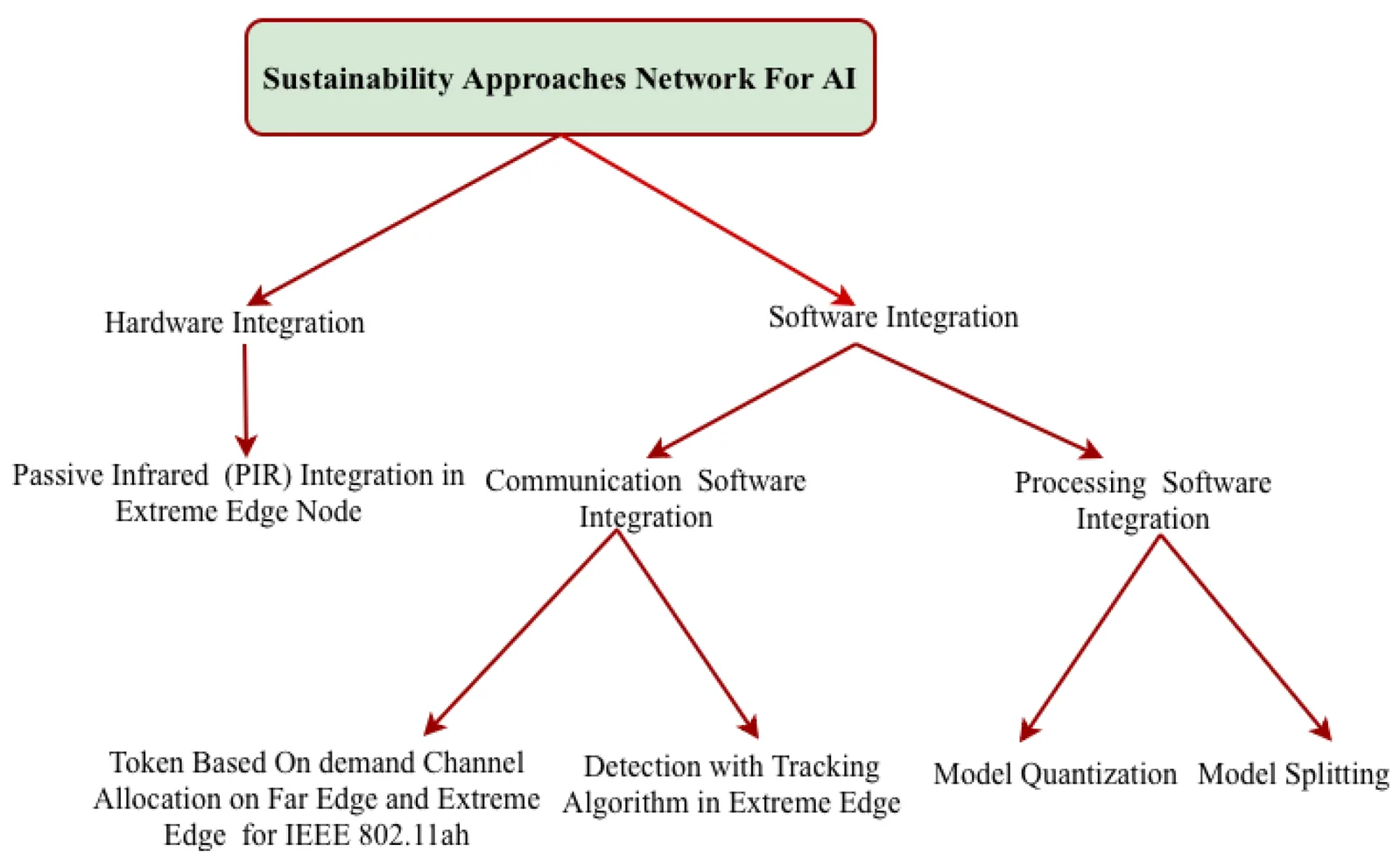
Sustainability Approaches Network for AI.

To support sustainable extreme-edge AI deployments in wildlife monitoring systems, several strategies shown in Figure 2 are implemented in our hierarchical network for AI to reduce energy consumption while maintaining reliable detection performance. First, hardware-assisted event triggering is employed using PIR sensors integrated into the processing board. The PIR sensors activate the camera and AI processing pipeline only when motion is detected, preventing continuous image processing and significantly reducing unnecessary energy consumption. Second, AI model quantization is applied to reduce the computational complexity and memory requirements of the deep learning models. Quantized models require fewer arithmetic operations and lower-precision computation, which reduces GPU or accelerator workload and power usage during inference. Third, efficient tracking algorithms are adapted to minimize redundant object detection computations. Instead of running full object detection on every frame, lightweight tracking algorithms maintain object identities across frames, thereby reducing the frequency of computationally intensive inference operations. Fourth, AI model splitting and collaborative inference are used to distribute the computational workload across multiple edge devices. In this approach, the backbone of the neural network extracts feature representations on one device, while the remaining neck and detection layers process the feature maps on another device. This distributed inference framework reduces computational load on individual devices and improves overall energy efficiency. Together, these techniques reduce redundant computation, minimize device power consumption, and enable the development of sustainable network AI systems that align with green energy and low-power edge computing principles.

### 4.1. Hardware Integration for Energy-Efficient Processing and Communication

**Figure 3:**
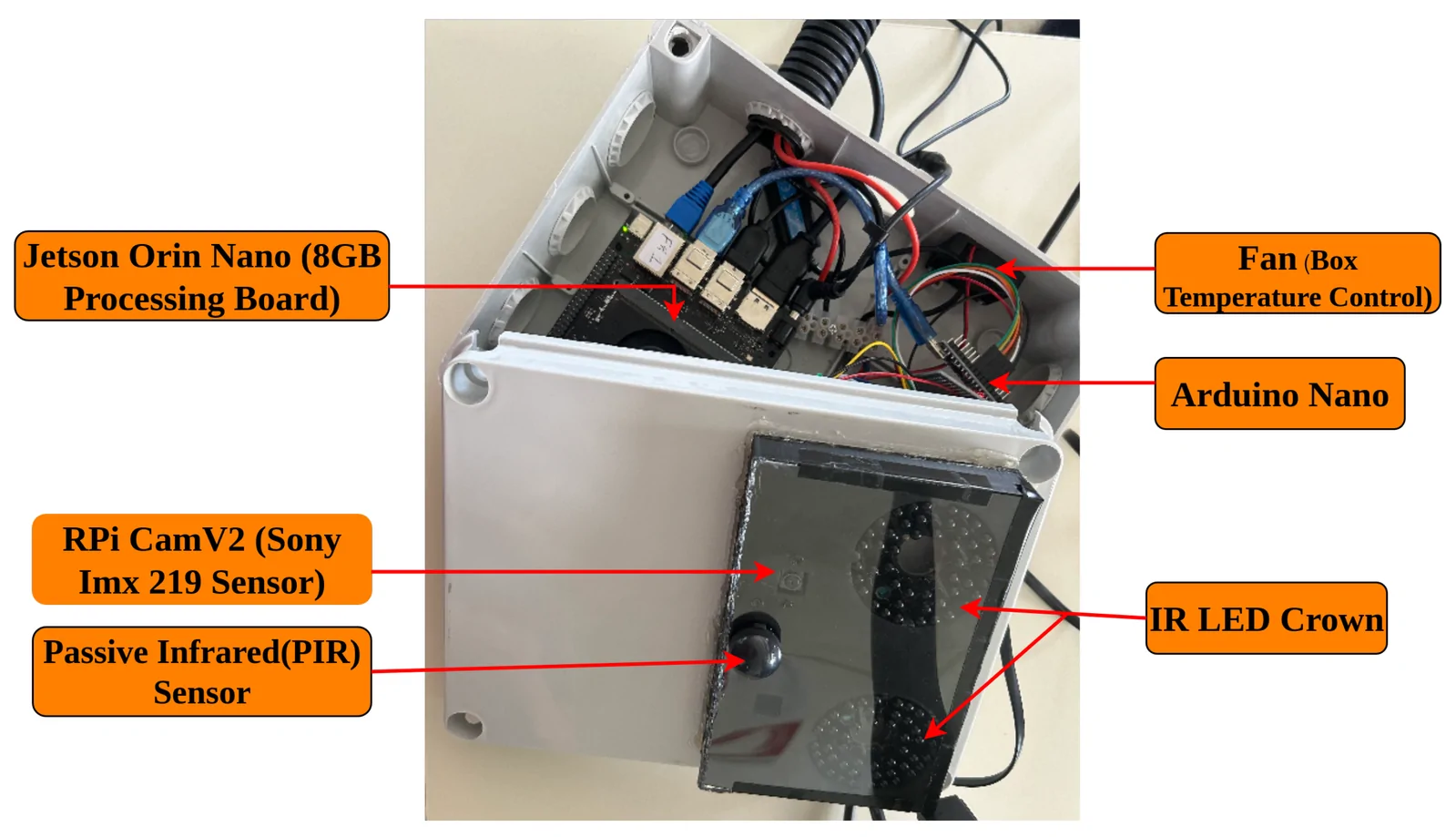
Internal Equipment of Extreme Edge Node.

**Figure 4:**
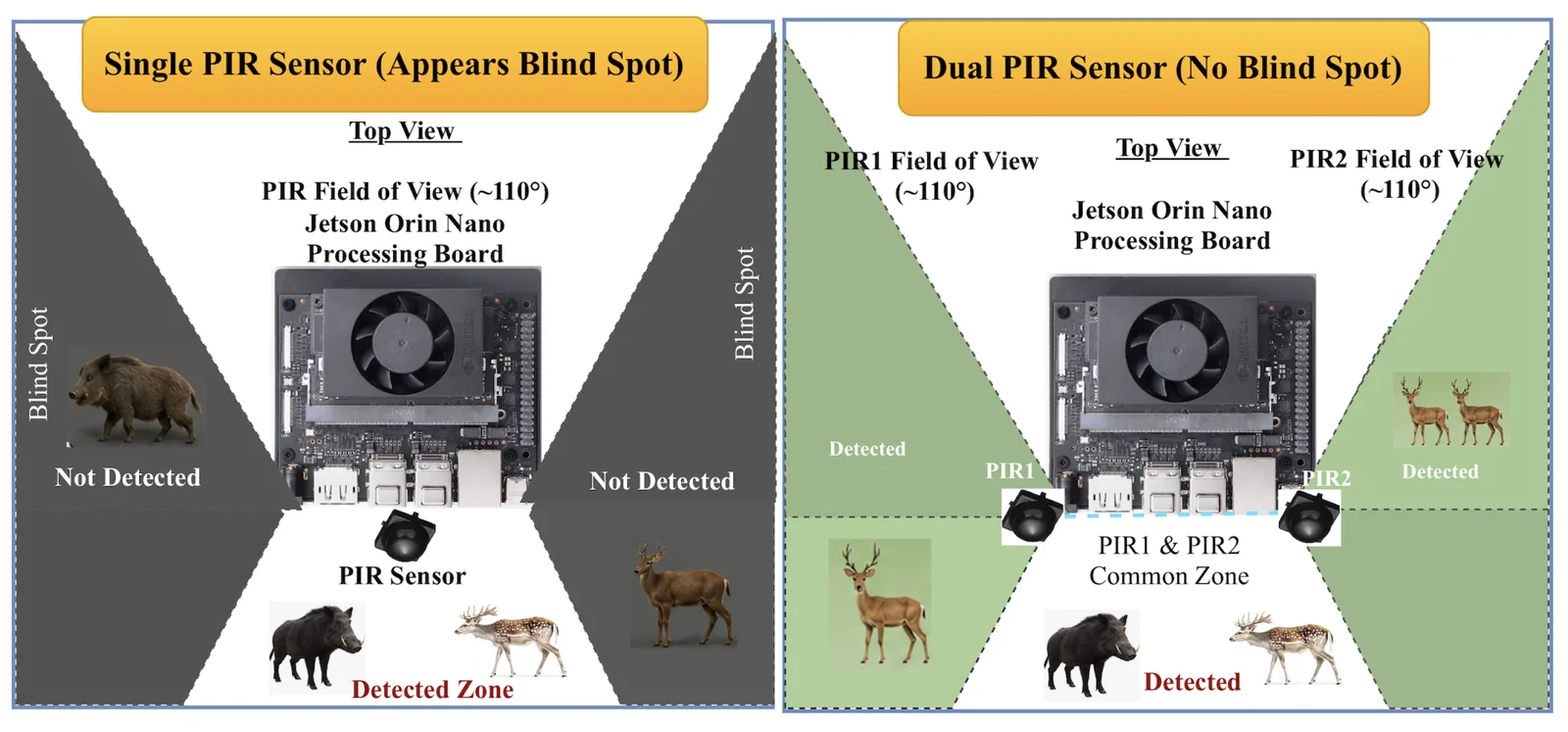
Comparison between single PIR and dual PIR integration. Single PIR sensor has limited field of view (∼110° creating blind spots (black spots)) on both sides. Dual PIR sensors provide wide and overlapping coverage, eliminating blind spots.

A PIR sensor is integrated into the extreme-edge node processing board based on the Jetson Orin Nano using an Arduino Nano microcontroller. The PIR sensor serves as a low-power trigger mechanism that controls the transition of the processing board between idle and active operating states. During periods of inactivity, the system remains in an energy-efficient idle state, while detected motion activates the monitoring and AI processing pipeline. However, a single PIR sensor has a limited field of view and detection range, which may create blind spots and result in missed motion events when animals approach from different directions. To improve detection reliability, a second PIR sensor was integrated into the processing board, as shown in Figure 3 and Figure 4. The dual-PIR configuration expands the sensing coverage area and increases the probability of detecting movement in wildlife monitoring environments.

**Figure 5:**
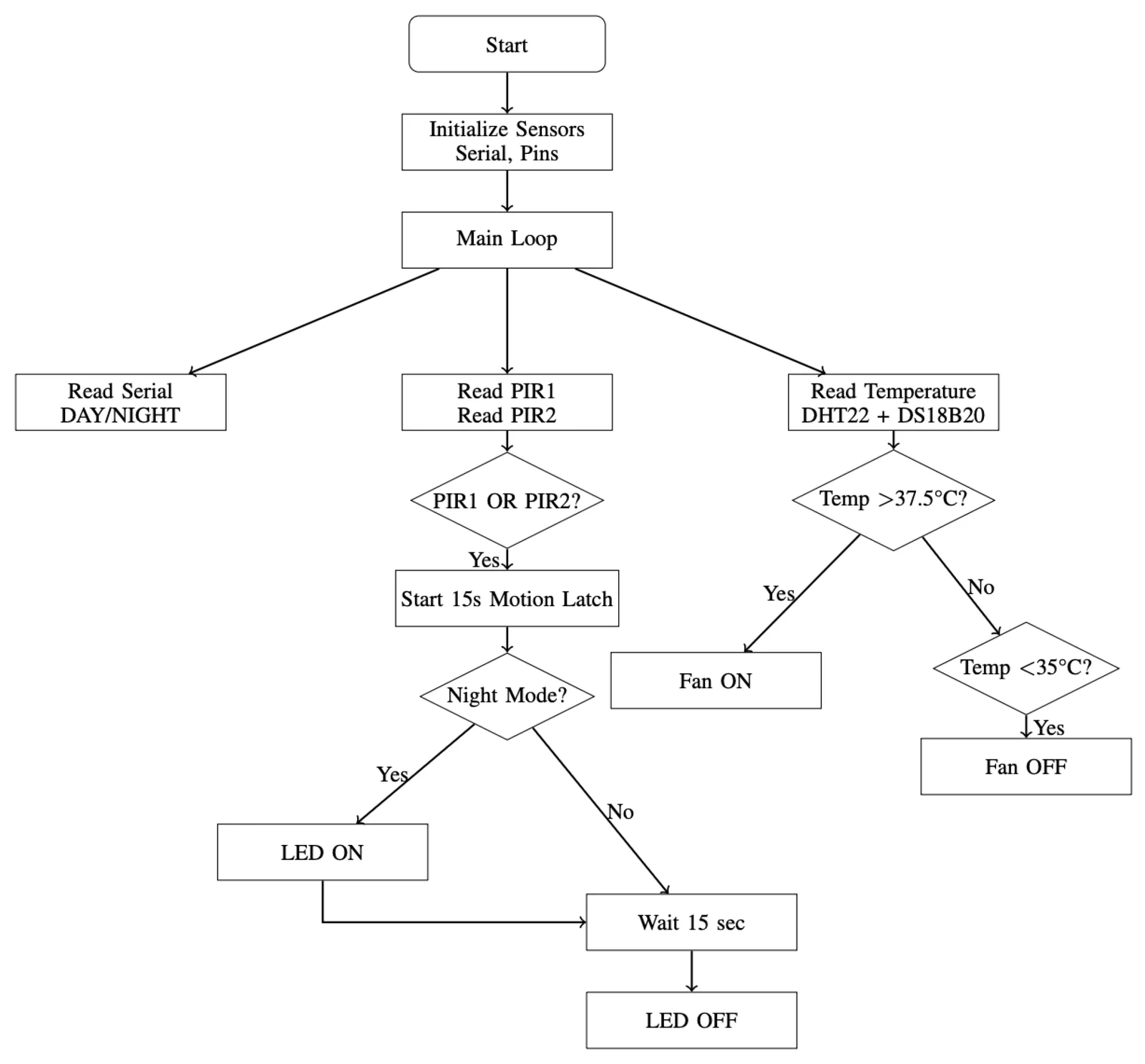
Workflow of the environmental monitoring and motion-triggered control system using dual PIR sensors.

As illustrated in Figure 5, the Arduino Nano continuously acquires motion data from both PIR sensors and environmental measurements from the DHT22 and DS18B20 sensors. When either PIR sensor detects motion, a 15-s latch mechanism maintains the trigger signal, ensuring stable activation of the processing board and preventing rapid repeated state transitions. During nighttime operation, detected motion activates LED illumination, while DAY and NIGHT commands received from the Jetson Orin Nano control the camera’s IR-cut filter for automatic switching between daytime and infrared imaging modes. The DS18B20 sensor also regulates a cooling fan to maintain safe operating temperatures. This integrated sensing and control framework enables reliable event-driven operation, reduces unnecessary power consumption, and supports autonomous wildlife monitoring at the extreme edge.

### 4.2. Software Integration for Energy-Efficient Processing and Communication

This section presents the software-level strategies adopted to improve energy efficiency in both processing and communication within the AP and extreme-edge nodes. The proposed approach focuses on reducing overall power consumption through optimized software integration and intelligent system operation.

At the communication level, power consumption is minimized by implementing an on-demand channel allocation mechanism. In this approach, communication is not performed continuously; instead, data transmission occurs only when required. This significantly reduces unnecessary channel usage and lowers energy consumption, especially in low-traffic scenarios.

At the processing level, energy efficiency is achieved through optimized edge AI deployment. The proposed system uses model optimization techniques such as model splitting and quantization to reduce computational complexity while maintaining acceptable inference accuracy. These techniques are preferred over traditional pruning methods to ensure a balance between performance and energy efficiency.

Furthermore, all processing tasks are executed at the edge node using the Jetson Orin Nano platform, reducing the need for continuous data transmission to centralized servers. This edge-centric approach not only minimizes communication overhead but also contributes to overall system sustainability.

Overall, the integration of energy-aware communication protocols and optimized AI processing enables efficient and sustainable operation of the proposed network architecture.

#### 4.2.1. Communication

Level Software Integration in AP and Extreme Edge Node

In this subsection, an energy-efficient communication framework is proposed for both the AP and extreme-edge nodes. The objective is to minimize power consumption by avoiding continuous communication and processing operations. Instead, a demand-driven mechanism is adopted, in which sensing, processing, and communication are activated only upon event detection.

At the extreme-edge node, a PIR-based event-triggering mechanism is used to initiate image acquisition and edge AI processing using the YOLOv8m model. Communication is performed only when relevant objects are detected, thereby reducing unnecessary data transmission. At the AP, a token-based channel allocation scheme is implemented to efficiently manage multiple edge nodes and ensure fair and collision-free communication.

The proposed approach significantly reduces idle listening, processing overhead, and communication energy consumption, making it suitable for sustainable and low-power IoT network deployments.

These two algorithms work together to enable sustainable, energy-efficient, and communication-aware operation in the proposed extreme-edge wildlife monitoring architecture. The first algorithm focuses on reducing unnecessary sensing, computation, and communication at the extreme-edge node, while the second algorithm manages communication access efficiently among multiple deployed nodes.

The on-demand energy-efficient communication at the extreme-edge node, presented in Algorithm 1, operates using an event-driven approach in which the extreme-edge node remains in a low-power sleep state by disabling energy-intensive components such as the camera, processing board, and IEEE 802.11ah communication interface. The PIR sensor continuously monitors motion while consuming minimal energy. Once motion is detected, the processing unit and camera are activated, and an image frame is captured for AI-based wildlife detection and tracking using YOLOv8m. If no wildlife is detected, the system immediately returns to a low-power state, avoiding unnecessary computation and communication. When wildlife is detected, the node activates the IEEE 802.11ah radio and initiates communication with the AP by generating a transmission token request. Only after receiving transmission permission does the node send detection metadata and tracking information. This event-driven workflow significantly reduces power consumption, bandwidth utilization, redundant frame transmission, and carbon emissions by ensuring that sensing, processing, and communication occur only when meaningful events are identified.

The token-based channel allocation at the IEEE 802.11ah AP algorithm in Algorithm 2 manages communication resources among multiple extreme-edge nodes to avoid network congestion and inefficient channel usage. When a node requests transmission access, the AP adds the node to a transmission queue. If communication resources are available, the AP allocates a channel and grants transmission permission; otherwise, the node remains queued until resources become available. After transmission completion, the allocated channel is released and reassigned to the next waiting node. This token-based scheduling mechanism ensures fair channel allocation, minimizes collisions, reduces unnecessary retransmissions, and improves network scalability in hostile environments where multiple extreme-edge wildlife monitoring nodes operate simultaneously. Together, these algorithms create a sustainable Network for AI architecture by jointly optimizing sensing, computation, communication, and  energy consumption across the deployed infrastructure.
**Algorithm 1** On-Demand Energy-Efficient Communication at extreme-edge Node  1:Initialize system  2:node_state ← SLEEP  3:Disable camera, processing unit, IEEE 802.11ah radio  4:**while** system_active **do**  5:    **if** PIR detects motion **then**  6:          Wake processing unit  7:          Activate camera  8:          frame ← Capture image  9:          detections ← Run YOLOv8m detection and tracking10:          **if** wildlife detected **then**11:               Activate IEEE 802.11ah radio12:               token ← Generate token13:               Send request to Access Point14:               Wait for channel allocation15:               **if** permission granted **then**16:                   Transmit detection metadata17:                   Transmit tracking information18:             **end if**19:          **end if**20:          Deactivate camera21:          Set processing unit to low-power mode22:          Disable IEEE 802.11ah radio23:      **end if**24:**end while**

**Algorithm 2** Token-Based Channel Allocation at IEEE 802.11ah Access Point
  1:Initialize AP  2:Create transmission queue  3:**while** network_active **do**  4:    **if** token request received **then**  5:        Enqueue node  6:        **if** channel available **then**  7:             node ← Dequeue  8:             Allocate channel  9:             Send transmission permission10:        **else**11:             Keep node in queue12:        **end if**13:    **end if**14:    **if** transmission completed **then**15:        Release channel16:        **if** queue not empty **then**17:             next_node ← Dequeue18:             Allocate channel19:             Send permission20:        **end if**21:    **end if**22:
**end while**



#### 4.2.2. Detection and Tracking-Based Communication Optimization

https://app.roboflow.com/auhidur-rahman/european-animal-detection/1 (accessed on 15 December 2024) The integration of object detection with tracking significantly reduces communication overhead compared with detection-only approaches. In conventional detection-based systems, image data or detection results are often transmitted for every frame, resulting in excessive data offloading and increased energy consumption. In contrast, the proposed system combines object detection with tracking to minimize redundant transmissions. Specifically, a Kalman filter–based tracking approach is employed to maintain object trajectories across frames. To improve robustness under challenging conditions such as occlusion caused by vegetation and color similarity, additional appearance-based features are incorporated using local binary patterns (LBP) and the hue-saturation-value (HSV) color space, as explored in our previous work [38,39]. With this framework, data transmission occurs only once when an object is initially detected. In subsequent frames, tracking is used instead of repeated detection and communication. This significantly reduces redundant data transmission and lowers the frequency of communication. Such an approach is particularly advantageous in energy-constrained edge environments, where minimizing communication overhead directly contributes to reduced power consumption, enhanced system efficiency, and extended operational lifetime.

### 4.3. Processing Level Software Integration in Extreme Edge Nodes

For animal detection, we trained a custom YOLOv8m model using our European Animal Detection Dataset https://app.roboflow.com/auhidur-rahman/european-animal-detection/1 (accessed on 15 December 2024) [40], which consists of five classes: fallow deer fawn, fallow deer female, and fallow deer male, wild boar, and wolf. Although all five classes represent distinct categories in the dataset, three of them specifically refer to different types of fallow deer. After training the model, it achieved a mean Average Precision (mAP) of 92.3%. This trained PyTorch-based YOLOv8m model is deployed on each smart pole for real-time animal detection. The dataset size, construction process, class distribution, model training procedure, and comparative analysis of model performance were presented in our previous work [3]. The deployments leverages the ultralytics:latest-jetson-jetpack6 Docker image, which provides pre installed libraries and dependencies necessary for vision task. The software stack includes CUDA (12.6.68), cuDNN (9.0.3), TensorRT (10.3.0), OpenCV (4.11.0), PyTorch (2.5.0a0+872d972e41.nv24.08), and Python (3.10.12), running on JetPack 6.2 with support for YOLOv8 (8.3.74). The Docker container image requires additional software for camera access and gRPC communication. Specifi- cally, GStreamer must be installed to enable OpenCV-based camera access, and grpcio (1.62.1) along with grpcio-tools (1.62.1) are needed for gRPC communication.

#### 4.3.1. Model Optimization for Energy-Efficient Edge Processing

**Figure 6:**
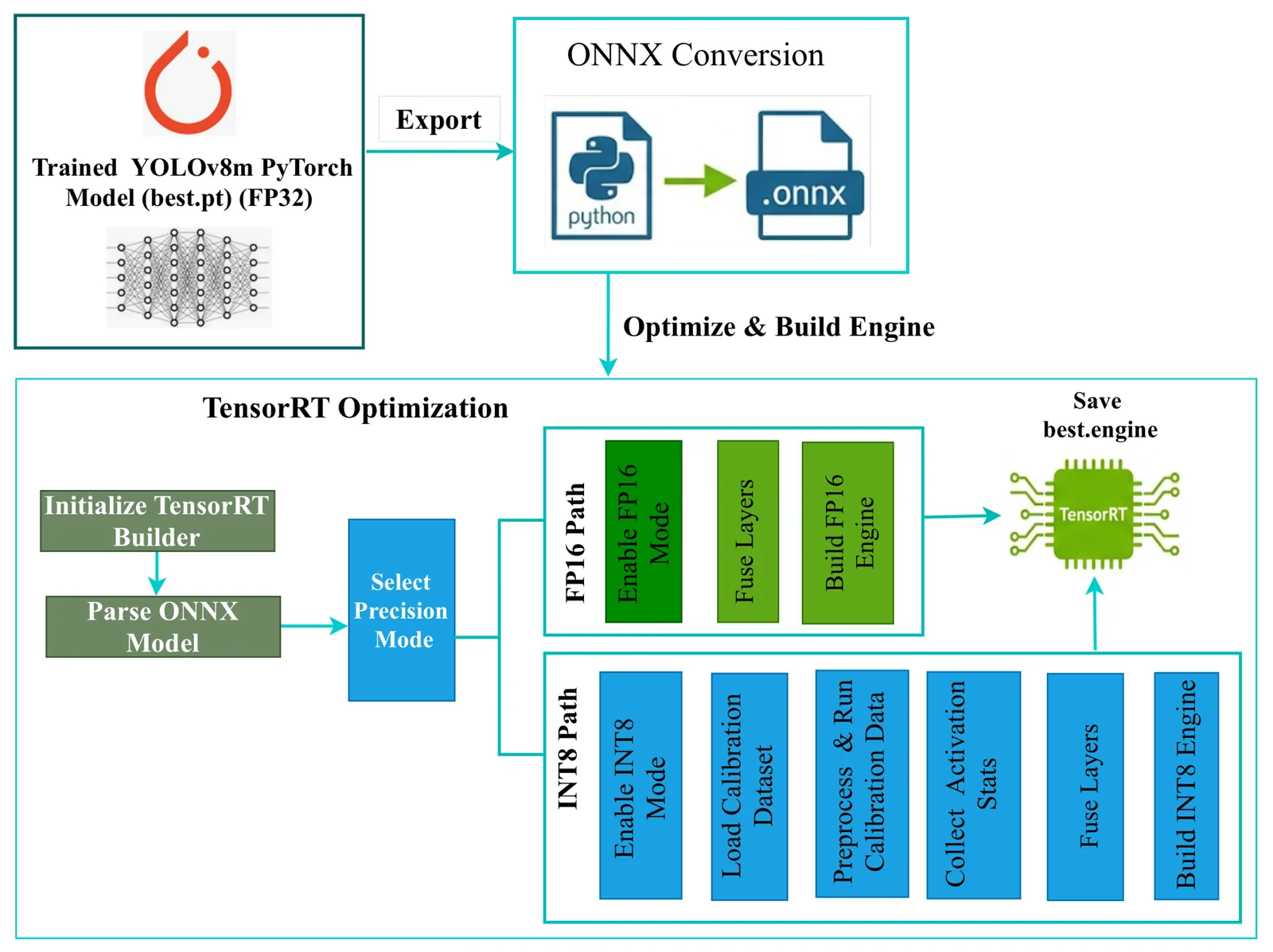
Model Quantization: FP32 Precision to FP16 and INT8 Precison Model.

The trained YOLOv8m model (best.pt) was optimized for deployment on the NVIDIA Jetson Orin Nano using TensorRT 10.2 through FP16 and INT8 quantization workflows. The overall optimization pipeline consists of model export, precision conversion, TensorRT engine generation, and deployment on the extreme-edge AI node. Initially, the trained PyTorch FP32 model (best.pt) was exported into the ONNX (Open Neural Network Exchange) format to provide interoperability between the training framework and TensorRT optimization engine. The ONNX model serves as the intermediate representation for subsequent TensorRT-based optimization and inference acceleration. For FP16 optimization, TensorRT converts the FP32 floating-point operations into half-precision FP16 computations supported by the NVIDIA Jetson Orin Nano GPU architecture. During this process, TensorRT performs layer fusion, kernel optimization, memory optimization, and mixed-precision scheduling to generate the optimized FP16 TensorRT engine (best.engine). The FP16 workflow reduces memory usage and computational complexity while maintaining detection accuracy close to that of the original FP32 model. Since FP16 does not require calibration, the conversion process is relatively straightforward and directly supported by TensorRT. In contrast, INT8 optimization requires an additional calibration stage. After exporting the FP32 model to ONNX format, TensorRT performs post-training quantization (PTQ) using an entropy-based calibration mechanism. A representative calibration dataset containing wildlife images under diverse environmental conditions was used to estimate the activation dynamic ranges of the neural network layers. During calibration, TensorRT analyzes the distribution of intermediate activations and computes appropriate scaling factors to map floating-point values into 8-bit integer representations. Based on the calibration results, TensorRT generates an INT8 calibration cache and constructs the optimized INT8 TensorRT engine. Compared with FP16 inference, INT8 quantization significantly reduces memory bandwidth requirements, arithmetic complexity, and power consumption. In the proposed framework, the YOLOv8m model is optimized by converting full-precision (FP32) computations to lower-precision formats such as FP16 and INT8, as shown in Figure 6.

Quantization reduces the numerical precision of model weights and activations, thereby decreasing computational complexity and memory usage. Compared with FP32, FP16 reduces memory bandwidth requirements and accelerates computation on supported hardware without requiring calibration. INT8 further improves efficiency by enabling faster arithmetic operations and significantly reducing energy consumption through lower bit-width representations.

The total power consumption of a deep learning inference system can be approximated as:

(1) $$P_{\mathrm{total}}=P_{\mathrm{compute}}+P_{\mathrm{memory}}$$

The computational power is proportional to the number of operations and the energy per operation:

(2) $$P_{\mathrm{compute}}\propto N_{\mathrm{ops}}\cdot E_{\mathrm{per}-\mathrm{op}}$$

By reducing precision from FP32 to INT8, the energy per operation significantly decreases:

(3) $$E_{\mathrm{INT}8}\ll E_{\mathrm{FP}32}$$

Thus, the computational power for INT8 inference becomes:

(4) $$P_{\mathrm{compute},\mathrm{INT}8}=N_{\mathrm{ops}}\cdot E_{\mathrm{INT}8}$$

Similarly, memory power depends on the volume of data transferred:

(5) $$P_{\mathrm{memory}}\propto D\cdot E_{\mathrm{access}}$$

Since INT8 uses 8-bit representations compared to 32-bit in FP32:

(6) $$D_{\mathrm{INT}8}=\frac{1}{4}D_{\mathrm{FP}32}$$

Therefore, the memory power consumption is reduced as:

(7) $$P_{\mathrm{memory},\mathrm{INT}8}\approx \frac{1}{4}P_{\mathrm{memory},\mathrm{FP}32}$$

Combining both components, the total power for INT8 inference can be expressed as:

(8) $$P_{\mathrm{INT}8}=N_{\mathrm{ops}}\cdot E_{\mathrm{INT}8}+\frac{1}{4}D_{\mathrm{FP}32}\cdot E_{\mathrm{access}}$$

In comparison, the FP32 power consumption is given by:

(9) $$P_{\mathrm{FP}32}=N_{\mathrm{ops}}\cdot E_{\mathrm{FP}32}+D_{\mathrm{FP}32}\cdot E_{\mathrm{access}}$$

The reduction in power consumption from FP32 to INT8 arises from two key factors: (i) reduced computational energy due to lower precision arithmetic, and (ii) reduced memory bandwidth requirements, as INT8 representations require four times fewer bits than FP32.

Using quantization, the proposed system achieves efficient edge AI processing with lower memory usage and minimized power consumption, making it highly suitable for energy-constrained edge deployments.

#### 4.3.2. Model Splitting for Distributed Energy-Efficient Inference

Manual Layer Index-Based Splitting

**Figure 7:**
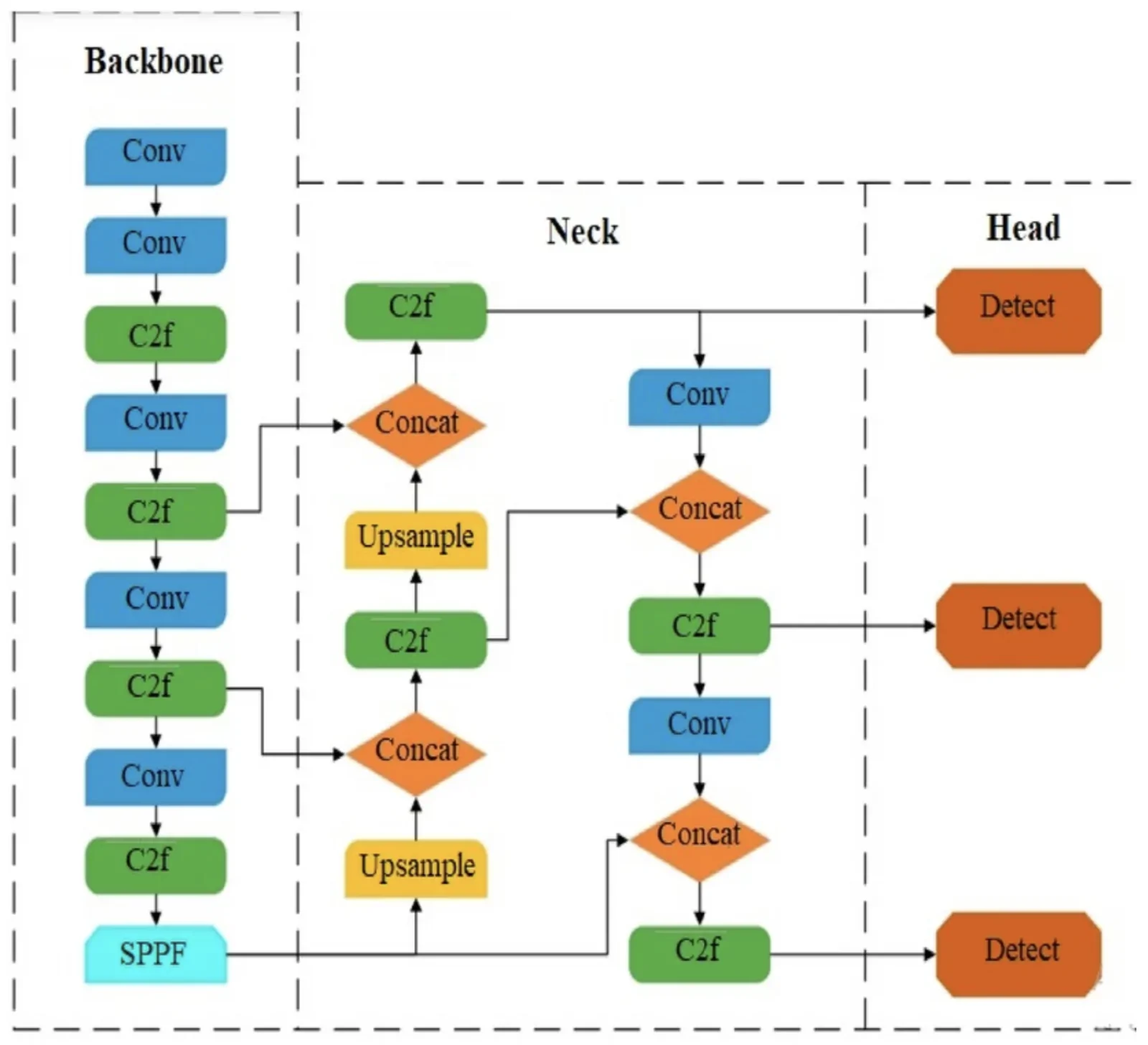
YOLOv8 Layer Architecture for Model splitting into Backbone and Neck_Head.

To further reduce computational load and power consumption at the extreme-edge node, a model splitting strategy is adopted in the proposed system. The YOLOv8m model, consisting of 22 layers as shown in Figure 7, is partitioned into two components: the backbone network (layers 0–9) and the neck-head network (layers 10–22) using the manual layer-based splitting approach shown in Algorithm 3.
**Algorithm 3** Manual Layer Index-Based Splitting of YOLO Model**Require:** 
Trained YOLO model file best.pt, split indices $\left(i_{start},i_{end}\right)$**Ensure:** 
Saved submodels backbone.pt, neck_head.pt  1:**procedure** Manual_Split_YOLO($best\_model\_path,i_{start},i_{end}$)  2:    full_model←YOLO(best_model_path).model  3:    layers_list←list(full_model.model)  4:                                                                                  ▹ Split model layers by indices  5:    $backbone\_layers\leftarrow layers\_list[0:i_{start}]$  6:    $neck\_head\_layers\leftarrow layers\_list[i_{start}:i_{end}]$  7:                                                      ▹ Wrap submodules for independent inference  8:    Define class **WrappedModule(submodel)**:  9:         forward(x)→submodel(x)10:                                                                                           ▹ Save the two modules11:    torch.save(backbone_module,“backbone.pt”)12:    torch.save(neck_head_module,“neck_head.pt”)13:    **print** (“Manual split completed successfully.”)14:**end procedure**

Following the partitioning process, the resulting models, backbone.pt and neck_head.pt, are validated through a series of tests, including sanity checks, numerical validation, numerical integrity verification, and functional equivalence testing to ensure consistency with the original model. The backbone model (backbone.pt) is deployed on the extreme-edge node, where it performs feature extraction and generates intermediate feature maps. These feature tensors ($P_{3}$, $P_{4}$, and $P_{5}$) are then converted to FP16 precision to reduce data size before being offloaded. The processed tensors are transmitted to the edge server using a lightweight communication protocol based on gRPC, as described in our previous work [31]. At the edge server, the neck-head model (neck_head.pt) completes the remaining inference tasks, including feature aggregation and object detection. This distributed inference architecture shifts computationally intensive operations away from the extreme-edge node, significantly reducing its processing burden and energy consumption. Moreover, transmitting compact intermediate feature tensors instead of full-resolution images substantially reduces communication bandwidth requirements. This reduction in data transfer further contributes to overall energy efficiency, particularly in bandwidth-constrained environments. Overall, the proposed model splitting and distributed inference framework enables an effective balance between computation and communication, resulting in improved system performance and reduced power consumption in energy-constrained edge deployments.

## 5. Solar Panel Capacity Calculation and Placement for Green Energy Production

In this section, we develop a generalized model for solar panel power capacity, battery sizing, and maximum power point tracking (MPPT) charge controller selection. Subsequently, we describe how green-energy-based equipment for the AP and extreme-edge node is integrated based on the calculated load requirements.

### 5.1. Generalized Solar Capacity, Battery Capacity and MPPT Controller Selection Model

The total daily energy consumption of the system is modeled by considering both continuously operating components and event-driven components. The generalized daily energy consumption is expressed as:

(10) $$E_{\mathrm{daily}}=\sum_{i=1}^{N_{c}}P_{c,i}T_{c}+\sum_{j=1}^{N_{e}}P_{e,j}T_{e,j}$$

where $P_{c,i}$ represents power consumption of continuously operating component *i* (W), $T_{c}$ is continuous operating duration (typically 24 h/day), $P_{e,j}$ represents power consumption of event-driven component *j* (W), $T_{e,j}$ is total active duration of event-driven component *j* (h/day).

For event-driven operation:

(11) $$T_{e,j}=N_{j}\times t_{j}$$

where $N_{j}$ and $t_{j}$ represents number of events per day and time duration per event, respectively.

Substituting:

(12) $$E_{\mathrm{daily}}=\sum_{i=1}^{N_{c}}P_{c,i}\left(24\right)+\sum_{j=1}^{N_{e}}P_{e,j}\left(N_{j}t_{j}\right)$$

Solar Panel Capacity Design:

The required solar panel capacity is determined from the daily energy consumption of the system and the available peak sunlight duration. The required solar array capacity is expressed as:

(13) $$P_{\mathrm{solar}}=\frac{E_{\mathrm{daily}}}{PSH\times \eta_{\mathrm{sys}}}$$

where $E_{\mathrm{daily}}$ represents the total daily energy consumption (Wh/day), PSH denotes the peak sun hours (hours/day), and $\eta_{\mathrm{sys}}$ represents the overall system efficiency considering MPPT efficiency, wiring losses, battery losses, and environmental factors.

To account for uncertainties such as weather variation, panel aging, dust accumulation, and additional system losses, a safety margin factor is introduced. Therefore, the recommended solar panel capacity is calculated as:

(14) $$P_{\mathrm{solar},\mathrm{recommended}}=P_{\mathrm{solar}}\times SF_{s}$$

where $SF_{s}$ is the solar oversizing factor, typically ranging between 1.2 and 1.5.

### 5.2. Battery Capacity Design

The required battery capacity is determined based on the daily energy consumption and the desired autonomy period. The battery energy requirement is expressed as:

(15) $$E_{\mathrm{battery}}=\frac{E_{\mathrm{daily}}\cdot D}{\eta_{b}\cdot DoD}$$

where $E_{\mathrm{daily}}$ is the daily energy consumption, *D* is the number of autonomy days, $\eta_{b}$ is the battery efficiency, and DoD is the allowable depth of discharge.

The battery capacity in ampere-hours (Ah) is given by:

(16) $$C_{\mathrm{Ah}}=\frac{E_{\mathrm{battery}}}{V_{\mathrm{system}}}$$

MPPT Charge Controller Sizing

The MPPT charge controller current rating is determined based on the total solar power input and the system battery voltage.

(17) $$I_{\mathrm{MPPT}}=\frac{P_{\mathrm{solar}}}{V_{\mathrm{battery}}}$$

To ensure safe operation under varying environmental conditions such as irradiance fluctuations, temperature effects, and transient current surges, a 25% safety margin is applied.

(18) $$I_{\mathrm{MPPT},\mathrm{recommended}}=1.25\times I_{\mathrm{MPPT}}$$

For systems with multiple solar panels, the total solar power is given by:

(19) $$P_{\mathrm{solar},\mathrm{total}}=N_{p}\times P_{\mathrm{panel}}$$

Thus, the MPPT current rating becomes:

(20) $$I_{\mathrm{MPPT}}=\frac{N_{p}\times P_{\mathrm{panel}}}{V_{\mathrm{battery}}}$$

where the 25% margin accounts for irradiance variations, temperature-dependent efficiency losses, and short-term current surges during operation.

### 5.3. Access Point Node Configuration and Network Model

The AP in the proposed network consists of a PoE switch, an IEEE 802.11ah transceiver for IoT connectivity, and a sub-6 GHz communication dish for midhaul communication, as shown in Figure 1. In the proposed network architecture, each AP communicates with multiple extreme-edge nodes, forming a point-to-multipoint topology. The IEEE 802.11ah transceiver enables low-power, long-range connectivity for edge devices, while the sub-6 GHz link provides reliable midhaul communication between the AP and the core network. During AP operation, the sub-6 GHz link and POE switch remain continuously active, whereas the IEEE 802.11ah transceiver is activated when a channel allocation request is received from an extreme-edge node. For this reason, two components of the AP are always on, and event-based activation of the IEEE 802.11ah transceiver will happen.

### 5.4. Load and Energy Requirement Analysis

The estimated power consumption of each component is summarized in Table 3. The values correspond to typical operational power rather than peak ratings.

The daily energy requirement is calculated using Equation (10).

(21) $$E_{\mathrm{daily}}=744+4\left(N_{j}t_{j}\right)$$

Assuming an average of $N_{j}=15$ transmission events per day and an average event duration of $t_{j}=10$ s (0.00278 h), the total active duration of the IEEE 802.11ah AP is

(22) $$T_{e}=N_{j}t_{j}=15\times 0.00278=0.0417\;h/\mathrm{day}$$

The event-driven energy consumption is therefore

(23) $$E_{\mathrm{event}}=4\times 0.0417=0.167\;\mathrm{Wh}/\mathrm{day}$$

Substituting into (21), the total daily energy consumption becomes

(24) $$E_{\mathrm{daily}}=744+0.167=744.17\;\mathrm{Wh}/\mathrm{day}$$

### 5.5. Solar Panel Capacity Calculation

Assuming an average peak sun hour value of PSH=5 h/day and an overall system efficiency of $\eta_{\mathrm{sys}}=0.8$, the minimum required solar panel capacity calculated using Equations (13) and (14)

(25) $$P_{\mathrm{solar}}=\frac{E_{\mathrm{daily}}}{PSH\times \eta_{\mathrm{sys}}}=\frac{744.17}{5\times 0.8}=186.04\;W$$

Considering a solar oversizing factor of $SF_{s}=1.3$ to account for weather variations, panel aging, dust accumulation, and other uncertainties, the recommended solar panel capacity becomes

(26) $$P_{\mathrm{solar},\mathrm{recommended}}=P_{\mathrm{solar}}\times SF_{s}=186.04\times 1.3=241.85\;W$$

Therefore, a commercially available 250 W solar panel is recommended for reliable operation of the proposed event-driven AP.

### 5.6. Battery Storage Requirement

Using the daily energy consumption of $E_{\mathrm{daily}}=744.17$ Wh/day from Equation (24), and assuming an autonomy period of D=2 days, a battery efficiency $\eta_{b}=0.9$, and allowable depth of discharge DoD=0.8, we substitute these values into Equation (15) to obtain the required battery energy as follows:

(27) $$E_{\mathrm{battery}}=\frac{744.17\times 2}{0.9\times 0.8}=2067.14\;\mathrm{Wh}$$

For a 12 V system, the required battery capacity is calculated using Equation (16)

(28) $$C_{\mathrm{Ah}}=\frac{2067.14}{12}=172.26\;\mathrm{Ah}$$

A 12 V, 200 Ah battery bank (4 × 50 Ah in parallel) combined with a 250 W solar panel provides sufficient energy to support a daily load of 744.17 Wh, although limited solar margin may affect performance under low irradiance conditions.

### 5.7. MPPT Charge Controller Sizing

The current rating of the MPPT charge controller is determined based on the total solar power input and the system battery voltage. For a 12 V battery system and a 250 W solar array, the MPPT current is given by Equation (17):

(29) $$I_{\mathrm{MPPT}}=\frac{P_{\mathrm{solar}}}{V_{\mathrm{battery}}}=\frac{250}{12}=20.83\;A$$

To ensure reliable operation under varying environmental conditions such as irradiance fluctuations, temperature variations, and transient current surges, a safety margin of 25% is applied, and the recommended MPPT current rating is given by Equation (18):

(30) $$I_{\mathrm{MPPT},\mathrm{recommended}}=1.25\times I_{\mathrm{MPPT}}=1.25\times 20.83=26.04\;A$$

Therefore, a commercially available 30 A MPPT charge controller is selected to ensure safe and reliable system operation.

### 5.8. Placement Considerations for Green Energy Production

To maximize solar energy harvesting, the photovoltaic panels should be oriented toward the south in Northern Hemisphere deployments and installed at a tilt angle approximately equal to the local latitude [41,42]. In addition, the installation site must be carefully selected to ensure that the panels are free from shading and other environmental obstructions throughout the day [43]. These placement strategies collectively improve overall system efficiency, enhance energy yield consistency, and ensure sustainable green energy production for continuous operation of the proposed network infrastructure.

### 5.9. Extreme-Edge Node Hardware Design, Load Calculation, Solar Panel Capacity, and Energy Requirement Analysis

**Figure 8:**
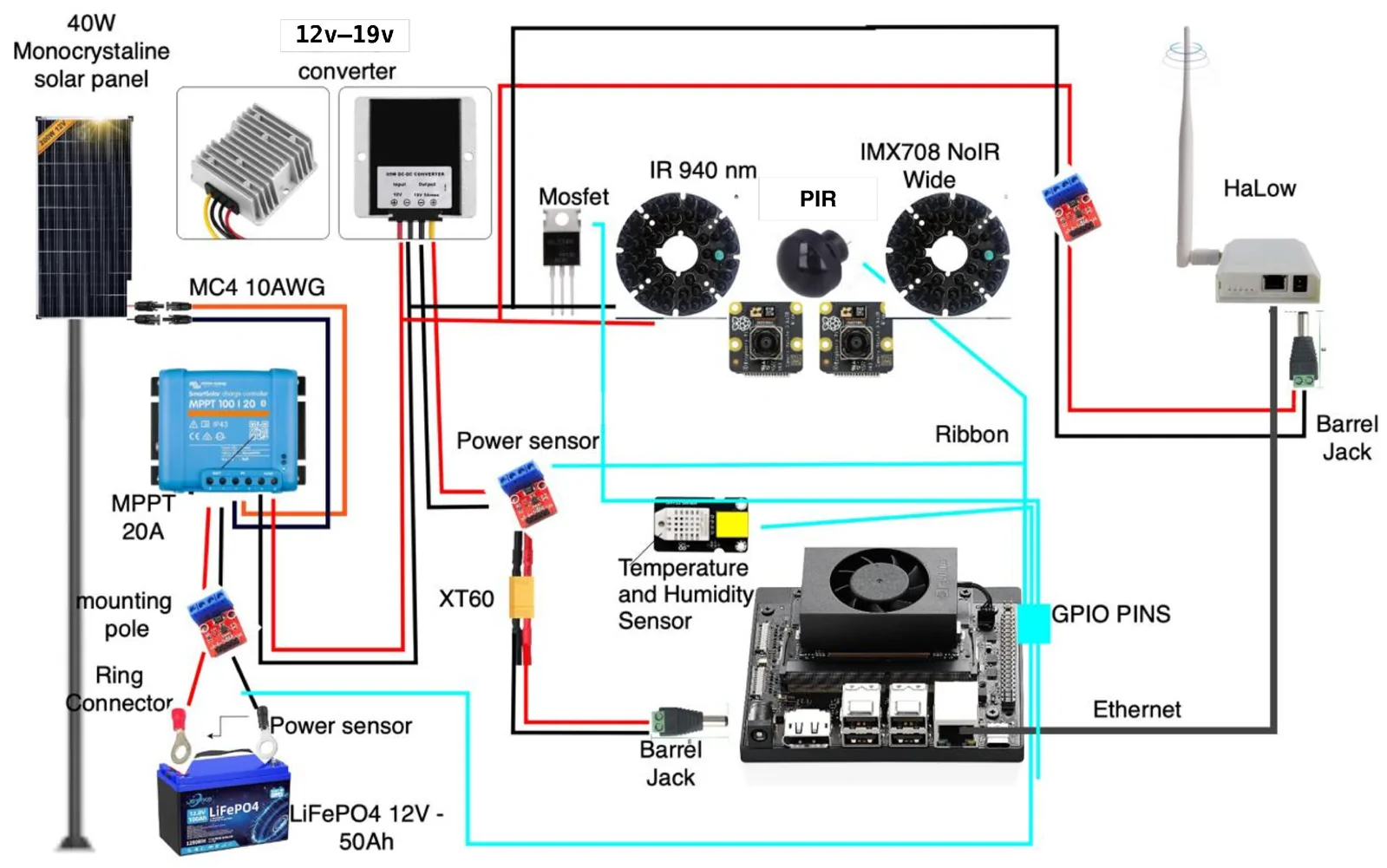
Hardware settings and internal device integration in extreme-edge node.

The extreme-edge node integrates a solar-powered energy system with intelligent sensing and edge AI processing. A 40 W monocrystalline solar panel is interfaced with a maximum power point tracking (MPPT) charge controller shown in Figure 8, which dynamically optimizes the operating voltage and current of the solar panel to maximize energy harvesting under varying irradiance and temperature conditions. The MPPT controller regulates the charging process of the 12 V LiFePO_4_ battery, ensuring efficient energy storage and protecting the battery from overcharging and deep discharge. The stored energy is subsequently regulated using a DC–DC converter to provide a stable and reliable supply voltage to the Jetson Orin Nano processing unit and associated peripherals.

The sensing and perception layer consists of NoIR camera modules connected via CSI interfaces, enabling both daytime and low-light monitoring with the support of 940 nm IR illumination. A PIR sensor is integrated through GPIO to enable event-driven operation. Specifically, the PIR sensor continuously monitors motion in the field of view and activates the camera, IR LEDs, and high-performance processing only when animal presence is detected. During idle periods, the system transitions to a low-power state, significantly reducing unnecessary energy consumption. A MOSFET-based switching mechanism is employed to control high-power peripherals such as IR LEDs, allowing selective activation based on environmental conditions and detection events. Additional environmental sensing is performed using temperature and humidity sensors, providing contextual data for wildlife monitoring.

For communication, an IEEE 802.11ah (Wi-Fi HaLow) module is interfaced via Ethernet, enabling long-range, low-power data transmission suitable for rural and remote deployments. Instead of continuous data streaming, the system adopts an event-driven communication strategy, transmitting only relevant time-series data (e.g., object class, tracking ID, confidence score, and environmental parameters), thereby minimizing bandwidth usage and communication energy. Power monitoring sensors are strategically deployed across the system to measure voltage, current, and power consumption at different stages, enabling real-time energy profiling and validation of MPPT efficiency and overall system optimization. This integrated hardware–software co-design enables a sustainable, energy-efficient far-edge node capable of autonomous operation in resource-constrained environments.

The itemized components in Table 4 sum to 42.9 W. This represents the calculated active-mode power consumption based on nominal component ratings and estimated system losses. The measured active power during field deployment ranges between 50–60 W due to additional transient loads, temperature-dependent losses, and peak current draw during simultaneous operation of all peripherals. Therefore, the design uses a conservative peak active power estimate of 55 W for solar sizing calculations, which is consistent with the measured worst-case operational power.

The extreme-edge node is further optimized at the system level through aggressive hardware and software power management techniques. These include reduced CPU core utilization, frequency scaling, PCIe power gating, and disabling unused peripherals such as the ISP, display, and CSI interfaces. As a result, the idle power consumption of the Jetson Orin Nano is reduced from approximately 10 W to 2.6 W, as measured using an external power analyzer. This optimization significantly enhances energy efficiency during non-event periods. The daily energy requirement for continuous 24-h operation is calculated as:

### 5.10. Energy Consumption and Solar Panel Sizing

Using Equation (12), the daily energy consumption of the proposed extreme-edge wildlife monitoring system is determined by considering both continuous and event-driven operation modes.

The system parameters are defined as follows: $P_{\mathrm{idle}}=2.6$ W, $P_{\mathrm{active}}=55$ W, average number of events per day N=15, average event duration $t_{e}=0.00278$ h (approximately 10 s), obtained from the four-week field deployment, peak sun hours PSH=5 h/day, system efficiency η=0.75, and safety factor γ=2.

The continuous energy consumption is given by:

(31) $$E_{c}=P_{\mathrm{idle}}\times 24=2.6\times 24=62.4\;\mathrm{Wh}/\mathrm{day}$$

The event-driven energy consumption is calculated as:

(32) $$E_{e}=P_{\mathrm{active}}\times \left(Nt_{e}\right)=55\times \left(15\times 0.00278\right)=2.29\;\mathrm{Wh}/\mathrm{day}$$

Thus, the total daily energy consumption is:

(33) $$E_{\mathrm{daily}}=E_{c}+E_{e}=62.4+2.29=64.69\;\mathrm{Wh}/\mathrm{day}$$

The required solar panel capacity is determined using Equation (13):

(34) $$P_{\mathrm{solar}}=\frac{E_{\mathrm{daily}}}{PSH\times \eta }=\frac{64.69}{5\times 0.75}=17.25\;W$$

To ensure reliable operation under environmental uncertainties, a safety factor γ is applied as in Equation (14):

(35) $$P_{\mathrm{solar},\mathrm{recommended}}=\gamma \times P_{\mathrm{solar}}=2\times 17.25=34.50\;W$$

Thus, a 40 W solar panel is sufficient to sustain continuous operation of the extreme edge node.

This demonstrates that, due to event-driven operation and aggressive power optimization, a 40 W solar panel is sufficient to sustain continuous operation of the extreme-edge node. In contrast, without such optimizations (idle power ≈10 W), the daily energy requirement would exceed 240 Wh/day, requiring a significantly larger solar panel (approximately 80–100 W) and making the system less practical for remote deployments. Therefore, the proposed architecture enables sustainable edge AI operation with minimal solar infrastructure, making it highly suitable for wildlife monitoring in off-grid environments.

### 5.11. Battery and MPPT Charge Controller Sizing

Using Equation (15), the required battery capacity is determined based on the daily energy consumption of the extreme-edge node.

For the proposed system, the parameters are defined as: $E_{\mathrm{daily}}=64.69$ Wh/day, autonomy days D=2, battery efficiency $\eta_{b}=0.9$, depth of discharge DoD=0.8, and system voltage $V_{\mathrm{system}}=12$ V.

The required battery energy capacity is given by:

(36) $$E_{\mathrm{battery}}=\frac{64.69\times 2}{0.9\times 0.8}=\frac{129.38}{0.72}=179.69\;\mathrm{Wh}$$

The corresponding battery capacity in ampere-hours is calculated using Equation (16):

(37) $$C_{\mathrm{Ah}}=\frac{179.69}{12}=14.97\;\mathrm{Ah}$$

Therefore, a commercially available 12 V, 20 Ah battery is selected to ensure reliable operation under practical conditions and provide additional design margin.

MPPT Charge Controller Sizing

Using Equation (17), the MPPT charge controller current is calculated based on the solar panel capacity $P_{\mathrm{solar}}=17.25$ W (from Equation (34)) and system voltage $V_{\mathrm{battery}}=12$ V:

(38) $$I_{\mathrm{MPPT}}=\frac{17.25}{12}=1.44\;A$$

Applying a 25% safety margin using Equation (18):

(39) $$I_{\mathrm{MPPT},\mathrm{recommended}}=1.25\times 1.44=1.80\;A$$

Therefore, a commercially available 5 A MPPT charge controller is recommended to ensure safe operation under transient and environmental variations.

## 6. Networking for Extreme-Edge AI: Working Principle

This section describes the integrated hardware–software architecture and operational workflow of the proposed extreme-edge AI network for efficient and energy-aware deployment. The system is designed to distribute intelligence across extreme-edge nodes while minimizing energy consumption, communication overhead, and latency.

**Figure 9:**
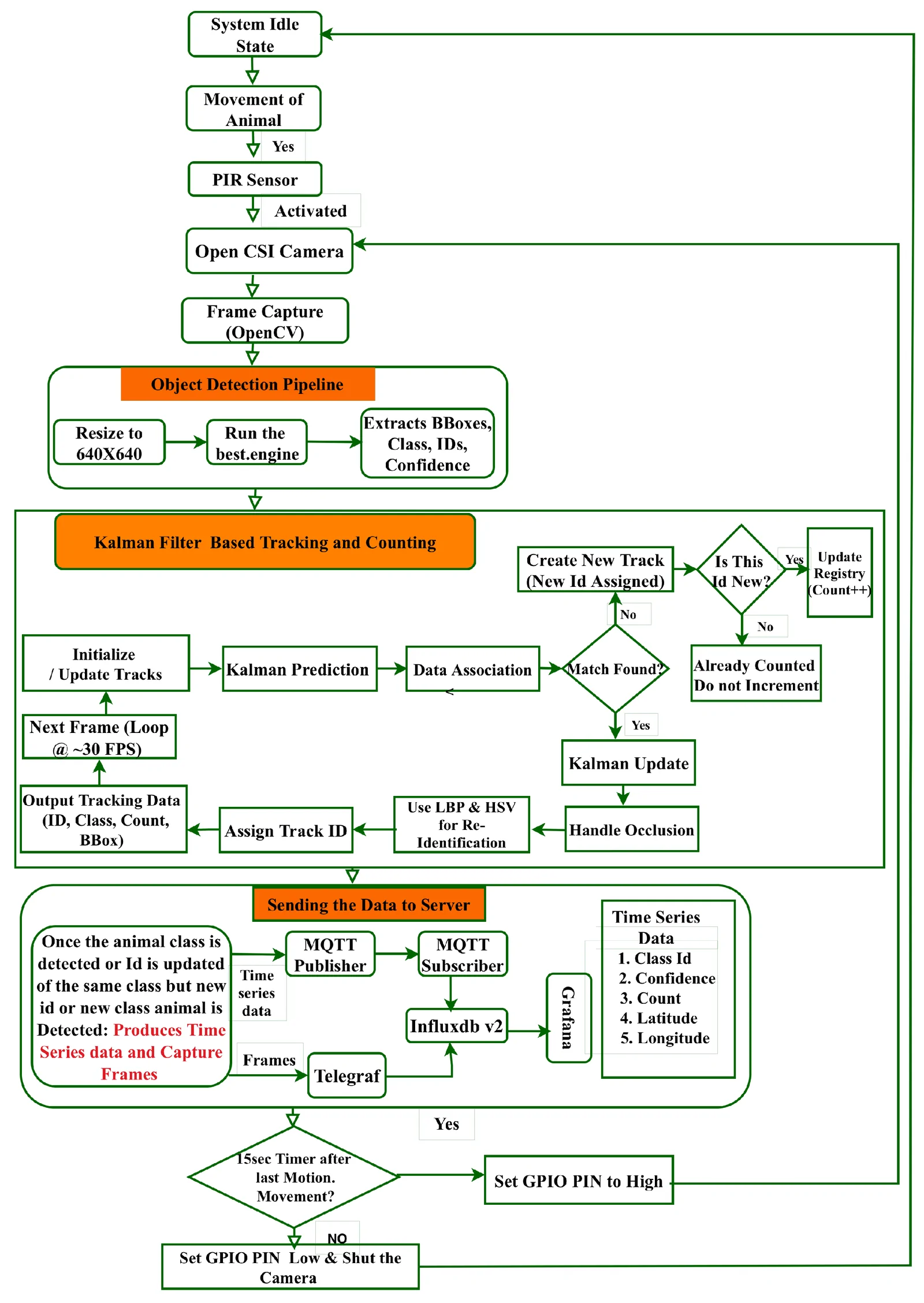
Sustainable Network for AI Working Flow Diagram.

At the extreme-edge node, a PIR sensor continuously monitors the camera’s field of view (FOV). When animal presence is detected, the PIR sensor activates the processing unit and camera module. The onboard processing device, based on the NVIDIA Jetson Orin Nano platform, captures image frames and performs real-time inference using a YOLOv8m model for object detection. Following detection, a Kalman filter-based tracking algorithm is employed to maintain temporal consistency and assign a unique track ID to each detected animal. To handle challenging scenarios such as occlusion caused by vegetation or overlapping animals, the system integrates appearance-based features using local binary patterns (LBP) and hue-saturation-value (HSV) representations. This enables robust re-identification and continuous tracking across frames at approximately 30 FPS, as shown in Figure 9. The system generates structured time-series data comprising object class labels, track IDs, confidence scores, and environmental parameters such as temperature and humidity collected from a DHT22 sensor. To ensure efficient wireless communication, the extreme-edge node participates in a token-based channel allocation mechanism over IEEE 802.11ah. The processing node transmits data only when it holds the communication token, ensuring collision-free and energy-efficient access to the network. To further reduce communication overhead, the system adopts an event-driven transmission strategy. For objects with the same track ID, data is transmitted only once unless a significant change is observed. This avoids redundant data transfer and minimizes bandwidth usage. Additionally, if no animal movement is detected for a predefined duration (e.g., 15 s), the processing unit transitions into a low-power idle state, thereby conserving energy in resource-constrained environments.

The generated time-series data is transmitted using the MQTT protocol from the extreme-edge node to the edge server for real-time data aggregation and analysis. Finally, the processed information is visualized through a Grafana dashboard, enabling continuous monitoring and actionable insights for wildlife tracking and rural safety applications.

## 7. Result Analysis and Discussion

In this section, we evaluate the sustainability of the proposed hierarchical edge AI network from the perspectives of computation, communication, and system operation. Sustainability is achieved through five complementary mechanisms. First, model optimization using FP16 and INT8 quantization reduces computational complexity, memory usage, and inference power consumption compared with FP32 execution. Second, PIR-based activation enables on-demand system operation by activating the camera and processing pipeline only when motion is detected, thereby minimizing idle energy consumption. Third, collaborative split-model inference is evaluated against full-model execution to assess its impact on power consumption and energy efficiency within the hierarchical edge architecture. Fourth, token-based channel allocation enables efficient event-driven communication, reducing channel contention, bandwidth utilization, and communication-related power consumption. Finally, tracking-assisted detection minimizes redundant processing and data transmission by reporting only newly observed object identities rather than frame-by-frame detections. Together, these computation- and communication-level sustainability mechanisms improve energy efficiency, reduce network overhead, and enhance the long-term operational sustainability of the proposed wildlife monitoring system.

### 7.1. Quantization Role for Achieving Sustainability of Extreme Edge AI

In this section, we evaluate the performance of the YOLOv8m architecture on the NVIDIA Jetson Orin Nano (8 GB) platform. The objective is to quantify the impact of quantization (FP32, FP16, and INT8) on inference throughput, power efficiency, memory residency, and detection accuracy during wildlife monitoring tasks. The experiments were conducted on a Jetson Orin Nano module configured in a 15 W power envelope (MAX-N mode). The software stack included JetPack 6.2, TensorRT 10.2.2, and CUDA 12.6. Profiling was performed using tegrastats, jtop, and trtexec to measure system power, GPU utilization, inference latency, and memory consumption.

#### 7.1.1. Inference Performance and Resource Utilization

Table 5 summarizes the performance across different precision levels. Reducing numerical precision significantly improves inference throughput while lowering GPU utilization and memory footprint. INT8 quantization achieves the highest efficiency, reaching 29.8 FPS with substantially reduced resource usage compared to FP32.

#### 7.1.2. Quantization Impact on Detection Accuracy (Lab Evaluation)

To evaluate the impact of quantization on detection accuracy, we constructed a European wildlife dataset comprising 10,000 annotated images across five classes: Fallow Deer Fawn, Fallow Deer Female, Fallow Deer Male, Wild Boar, and Wolf. For each class, 2000 images were collected to ensure balanced representation. The dataset was split into training (70%), validation (20%), and test (10%) sets, resulting in 7000 images for training, 2000 for validation, and 1000 for testing.

The YOLOv8m model was trained on the training set using FP32 precision. The trained FP32 weights were then quantized to FP16 and INT8 precision using TensorRT post-training quantization. All three precision variants (FP32, FP16, and INT8) were evaluated on the same held-out test dataset (1000 images) to ensure a fair comparison.

Table 6 presents the accuracy comparison across precision levels. The FP32 baseline achieves a mAP@0.5 of 92.3% on the test dataset. FP16 quantization shows negligible degradation (mAP = 92.1%), while INT8 quantization results in a moderate reduction to 89.7%, corresponding to a 2.6% absolute decrease in mAP. The accuracy drop is more pronounced for nighttime low-contrast images (91.8% to 88.4% for INT8) compared to daytime images (92.7% to 90.9% for INT8), consistent with the known sensitivity of INT8 quantization to low-contrast features.

Based on these lab results, INT8 quantization was selected for field deployment due to its superior throughput (29.8 FPS), lower power consumption (9.7 W), and acceptable accuracy trade-off (89.7% mAP).

#### 7.1.3. Field Deployment Accuracy of INT8 Model

To validate the real-world performance of the INT8 quantized model, we conducted field trials using video footage captured by the deployed system. The INT8 model was evaluated against ground truth (GT) annotations obtained from co-located trail cameras across seven video sequences covering both daytime and nighttime conditions. Table 7 presents the precision, recall, and average precision (AP) metrics for each video.

The INT8 model achieves a daytime average precision of 86.40% and a nighttime average precision of 79.37% in the field, with an overall mAP of 82.89%. The lower nighttime performance is primarily attributed to the 6.5 s Wild Boar video (AP = 52.35%), which featured challenging low-light conditions and occluded views. Excluding this outlier, the nighttime AP increases to 92.88%.

Under daytime conditions, Fallow Deer (Fawn, Female, and Male) achieve high detection rates with AP values of 92.86%, 92.10%, and 92.86%, respectively, while Wild Boar daytime AP is lower at 73.33%. In nighttime conditions, all available videos feature Wild Boar, with AP values ranging from 52.35% to 93.33%. The significant variation in Wild Boar nighttime performance is attributed to differences in illumination, occlusion, and camera angle across the video sequences. The lowest AP (52.35%) corresponds to a 6.5 s video with challenging low-light conditions and partial occlusion, while the higher AP values (93.33% and 90.95%) were achieved under better illumination with clearer animal visibility.

Compared with the FP32 model’s projected field performance (Table 8), the INT8 model shows a 2.6% absolute decrease in overall mAP (85.49% to 82.89%), which is consistent with the lab-measured degradation ratio. This confirms that INT8 quantization maintains acceptable detection performance in real-world conditions while providing significant computational and energy benefits.

#### 7.1.4. Projected Field Accuracy for FP32 Model

For comparison, Table 8 presents the projected field accuracy for the FP32 model, derived from the lab degradation ratios. The FP32 model is expected to achieve an overall field mAP of 85.49%, with daytime performance of 89.00% and nighttime performance of 81.97%.

#### 7.1.5. System-Level Power and Energy Measurements with INT8

##### Software vs. Hardware Power Measurements

In this work, two complementary power measurement approaches are employed:

- $P_{\mathrm{SW}}$ (Software Power): Measured using tegrastats and jtop, which report the Jetson Orin Nano’s on-chip power consumption. This value represents the power drawn by the Jetson’s CPU, GPU, and memory subsystems during inference.
- $P_{\mathrm{HW}}$ (Hardware Power): Measured using a Victron SmartSolar MPPT controller connected in series with the battery output. This value represents the total system power drawn from the battery, including the Jetson, camera module, IR LEDs, IEEE 802.11ah transceiver, sensors, MPPT controller quiescent power, DC-DC converter losses, and cabling losses.

The efficiency is calculated as the ratio of software power to hardware power:

(40) $$\mathrm{Efficiency}=\frac{P_{\mathrm{SW}}}{P_{\mathrm{HW}}}\times 100$$

The gap between $P_{\mathrm{SW}}$ and $P_{\mathrm{HW}}$ represents the power consumed by peripherals and system-level losses. A higher efficiency indicates better energy utilization and lower system losses.

To quantify the energy benefits of INT8 quantization in the deployed system, we measured the system-level power consumption under two scenarios: (i) continuous operation (without PIR) and (ii) event-driven operation (with PIR). Table 9 presents the measured power and daily energy consumption for the system running the INT8 model compared with the FP32 baseline.

The system running the INT8 model achieves a 39.30% reduction in continuous daily energy consumption (1320 → 801.24 Wh/day) compared with FP32. In the event-driven (with PIR) scenario, the INT8 system consumes 63.76 Wh/day (measured), representing a 95.17% reduction compared with FP32 continuous operation (1320 Wh/day). This measured value is slightly lower than the calculated estimate of 64.58 Wh/day (95.11%) obtained from field-observed averages in Section 7.2, with the difference attributed to real-world measurement variations and system efficiencies.

##### Derivation of Daily Energy Values

The “Without PIR” daily energy values in Table 9 are derived by scaling the full system active power ($P_{\mathrm{active},\mathrm{system}}=55$ W) by the ratio of hardware power for each precision relative to FP32:

(41) $$E_{\mathrm{without}\;\mathrm{PIR},\mathrm{precision}}=P_{\mathrm{active},\mathrm{system}}\times 24\times \left(\frac{P_{\mathrm{HW},\mathrm{precision}}}{P_{\mathrm{HW},\mathrm{FP}32}}\right)$$

where:

- $P_{\mathrm{active},\mathrm{system}}=55$ W is the measured peak power of the full system during continuous active operation (used consistently across Section 7.1.5 and Section 7.2).
- $P_{\mathrm{HW},\mathrm{precision}}$ is the hardware power measured at the MPPT output.
- $P_{\mathrm{HW},\mathrm{FP}32}=17.8$ W is the hardware power for FP32 precision.
- The scaling factor $\left(P_{\mathrm{HW},\mathrm{precision}}/P_{\mathrm{HW},\mathrm{FP}32}\right)$ accounts for the proportional reduction in total system power as the Jetson’s computation becomes more efficient with lower precision.
- 24 is the number of hours per day.

Table 10 provides the detailed calculation for each precision level. The scaling factor is simply the ratio of hardware power for each precision relative to FP32 (e.g., for INT8: 10.8/17.8=0.607).

For the “With PIR” scenario, the daily energy is dominated by idle power ($P_{\mathrm{idle}}=2.6$ W) with a small contribution from active event processing. The With PIR value (63.76 Wh/day for INT8) is measured directly from the deployed system during event-driven operation, incorporating all real-world losses and inefficiencies.

#### 7.1.6. Discussion

The INT8-quantized YOLOv8m model was selected for field deployment based on its superior lab performance: 4.7× higher throughput (29.8 FPS), 61% lower memory usage (1.2 GB), and 39% lower hardware power consumption (10.8 W), with only a 2.6% mAP reduction (92.3% to 89.7%). Field validation of the INT8 model confirms its real-world effectiveness, achieving an overall mAP of 82.89% (86.40% daytime, 79.37% nighttime), with precision consistently above 96%. The system-level power measurements show that INT8 deployment reduces continuous daily energy consumption by 39.30% (1320 → 801.24 Wh/day) compared with FP32, and when combined with event-driven PIR activation, achieves 63.76 Wh/day—a 95.17% reduction compared to FP32 continuous operation. These results demonstrate that INT8 quantization, validated through both lab and field evaluations, provides a favorable balance between detection accuracy and energy efficiency for long-term autonomous wildlife monitoring in remote extreme-edge environments.

### 7.2. Event-Driven vs. Continuous Surveillance: A Sustainability Analysis

In this section, we quantify the energy advantage of a PIR-assisted event-driven architecture compared with a traditional 24-h active surveillance model. In remote extreme-edge environments, constant monitoring is often unnecessary as wildlife activity is sporadic. By utilizing a PIR sensor as a hardware trigger, the system transitions from a low-power quiescent state to an active inference state only when thermal motion is detected.

To quantify the energy requirements ($E_{\mathrm{req}}$), we propose a dual-state power model. Using Table 11 and Equation (12), the total daily energy consumption ($E_{\mathrm{total}}$) is calculated as the sum of energy used during idle periods and active event processing using the general energy model described in Section 5.10.

During the four-week field deployment, the activity log recorded numerous wildlife-related events under diverse environmental and operating conditions. Based on these field observations, the average activity rate was approximately 15 events per day, while the mean event duration was approximately 10 s, calculated from the recorded activity durations. These field-observed averages were used as representative operating conditions for estimating the daily energy consumption of the event-driven system.

The energy saving is calculated as

(42) $$ES\left(\%\right)=\frac{E_{\mathrm{baseline}}-E_{\mathrm{optimized}}}{E_{\mathrm{baseline}}}\times 100$$

Using the energy values in Table 11, the corresponding reduction is

(43) $$ES\left(\%\right)=\frac{4752-232.5}{4752}\times 100=95.11\%.$$

Under the field-observed operating conditions, the proposed architecture is estimated to reduce daily energy consumption by approximately 95.11% compared with continuous operation. This value is calculated using the measured active and idle power levels together with the average activity rate and mean event duration observed during the four-week field deployment. Thus, the reported 95.11% represents an estimated daily energy reduction under representative field-observed operating conditions rather than a direct 24-h experimental measurement.

In Table 11, the calculated results indicate a significant improvement in energy efficiency when using the PIR-based event-driven system compared to continuous operation. In the without-PIR scenario, the system remains active for the full 86,400 s, resulting in high energy consumption of 4752 kJ due to constant operation at 55 W. In contrast, the PIR-enabled system is active for an estimated total of 150 s per day, based on the field-observed average of 15 events per day and a mean event duration of 10 s per event, and remains in low-power idle mode for the remaining duration.

The estimated event-driven energy consumption is obtained from the measured power values and the field-observed activity characteristics. Specifically, the active energy is calculated as 55×150=8250 J, while the idle energy is calculated as 2.6×86,250=224,250 J. Therefore, the total estimated daily energy consumption is 232,500 J, or 232.5 kJ (64.58 Wh/day).

For comparison, continuous operation at the measured active power of 55 W requires 4,752,000 J, or 4752 kJ (1320 Wh/day). Hence, the estimated energy saving under the field-observed average activity conditions is 4519.5 kJ/day, corresponding to a 95.11% reduction in daily energy consumption.

The actual daily energy consumption varies with wildlife activity. Days with fewer or shorter events will result in lower energy consumption, whereas days with more frequent or longer events will require more active processing energy. Therefore, the 95.11% value should be interpreted as a representative daily energy-saving estimate based on the average activity characteristics observed during the four-week deployment.

From a sustainability perspective, this reduction indicates the potential for lower carbon emissions and improved energy efficiency in continuous monitoring systems. Such event-driven architectures are therefore more suitable for long-term deployment in resource-constrained and environmentally conscious IoT applications.

### 7.3. Full vs. Split Inference Comparison for Sustainability

To enable distributed intelligence in the proposed extreme-edge architecture, the YOLOv8 object detection model is partitioned into two functional components: the backbone and neck-head. The backbone module is deployed on the extreme-edge device and is responsible for feature extraction from incoming visual data, while the neck-head module is executed on a secondary edge node to perform higher-level feature aggregation and object classification. The overall system power consumption is modeled as the sum of backbone processing power, neck-head processing power, communication overhead, and fixed peripheral power consumption, including sensors, camera module, MPPT controller, and power regulation units.

Although the split inference strategy introduces additional communication overhead due to feature map transmission between nodes, it enables workload distribution across multiple devices, reducing the computational burden on individual Jetson platforms. This distributed design improves resource utilization and provides a scalable framework for hierarchical edge deployment. However, the total system energy consumption depends on both computation and communication costs and therefore must be evaluated holistically rather than at the per-device level.

Table 12 presents a comparison of centralized full-model inference and the proposed split-inference architecture at different optimization stages. The full-model baseline deployed on a single Jetson Orin Nano exhibits a total power consumption of 14.8 W. The baseline full-model inference power measured in this experiment (14.8 W) is slightly higher than the FP32 figure reported in Table 5 (14.2 W). This minor variation (0.6 W) is within the expected range of measurement variability across different test runs, thermal conditions, and background system processes and does not affect the comparative conclusions drawn from our split inference analysis. When the model is partitioned into backbone and neck-head components across two Jetson devices, the total system power remains comparable (14.5 W), indicating that model splitting primarily redistributes computational load rather than directly reducing energy consumption.

However, when the proposed PIR-based event-driven mechanism is introduced, the system power consumption significantly decreases to 5.5 W due to reduced active inference time and increased idle periods. Further integration of INT8 quantization reduces computational overhead, resulting in a minimum observed system power consumption of 4.3 W. These results demonstrate that while split inference enhances scalability and resource distribution, the dominant contributors to energy efficiency are event-driven execution and model quantization.

### 7.4. Token-Based Channel Allocation for Sustainable Communication Compared with TWT Baseline Communication

The proposed system performs object detection and tracking on video streams and generates structured IoT packets in the format <track_id, class_name, confidence, temperature, humidity>. Energy consumption is measured using an INA219 power monitoring module connected in series with the Jetson power supply. Two operational states are considered: idle (no detection/communication) and active (detection with token-based transmission over IEEE 802.11ah).

The instantaneous power and energy are given by:

$$P=V\times I,\;E=P_{avg}\times T$$

Communication-related energy is isolated as:

$$P_{comm}=P_{active}-P_{idle},\;E_{event}=P_{comm}\times T$$

#### 7.4.1. Experimental Dataset and Statistical Characterization for Channel Allocation

To ensure statistical robustness, the four-week field deployment recorded numerous wildlife-related activities under diverse environmental and operating conditions. The complete activity logs collected during this period indicated an average activity rate of approximately 15 events per day, with a mean event duration of approximately 10 s. These field-observed activity characteristics were used as representative operating conditions for the daily energy estimation presented in Section 7.2. For the detailed detection and tracking evaluation, 24 representative events were subsequently selected from the activities recorded during the four-week deployment. The selected events covered five wildlife classes (Wild Boar, Wolf, Fallow Deer Male, Fallow Deer Female, and Fallow Deer Fawn), with 4–5 repetitions per event type, and included diverse operating conditions such as day/night operation, 1–5 animals per frame, and event durations ranging from 5 to 45 s. These 24 representative events were used for the comparative detection and tracking experiments and their associated statistical characterization, rather than representing the total number of wildlife activities recorded during the deployment. Results are reported as mean ± 95% CI. Table 13 summarizes packet generation and energy consumption.

The aggregated analysis in Table 13 confirms consistent energy reduction (28.6% based on the adopted α=1.4 overhead factor). The sensitivity analysis (Table 14) shows that savings range from 29.2% to 45.1% when α varies from 1.2 to 1.6, confirming the robustness of the proposed mechanism. A paired *t*-test confirms statistical significance (p<0.01).

#### 7.4.2. Baseline Comparison and α Justification

For baseline comparison, a TWT-based IEEE 802.11ah system was experimentally measured using Newracom NRC7292 modules. The measured overhead factor under high traffic (15–20 events/hour) was α=1.42±0.10 Table 15, consistent with prior IEEE 802.11ah MAC characterization [44]. We adopt α=1.4 as a conservative estimate.

The baseline energy is modeled as $E_{baseline}=1.4\times E_{proposed}$.

##### Sensitivity Analysis

Varying α from 1.2 to 1.6 (Table 14) indicates that the proposed mechanism consistently outperforms TWT (29.2–45.1% savings), confirming robustness.

#### 7.4.3. Discussion

The token-based mechanism eliminates contention overhead by dynamically allocating channel access when events occur, avoiding TWT’s scheduled wake-up inefficiencies for bursty traffic. INA219 measurements indicate that energy consumption correlates with packet transmissions. Statistical characterization (24 events, 95% CI, paired *t*-test, sensitivity analysis) provides strong evidence that observed savings are genuine and reproducible.

### 7.5. Detection-Only Versus Detection with Tracking for Sustainable Communication: Experimental Results

This section evaluates how integrating object tracking with detection improves sustainability by reducing communication overhead through fewer packet transmissions.

#### 7.5.1. Experimental Dataset and Statistical Characterization for Detection and Tracking

A comparative study was conducted between (i) detection-only and (ii) detection with tracking using the representative event set described in the previous subsection, covering five wildlife classes (Wild Boar, Wolf, Fallow Deer Male, Fallow Deer Female, Fallow Deer Fawn) under diverse operating conditions. Results are reported as mean ± 95% CI.

With detection-only processing, every frame containing an animal generates a packet transmission, leading to duplicate counting (e.g., 384 detections for 2 wild boars). In contrast, tracking assigns a persistent ID per animal, transmitting only unique occurrences (e.g., 2 packets for 2 wild boars).

The communication power is:

$$P_{\mathrm{comm}}=N_{\mathrm{pkt}}\times P_{\mathrm{pkt}},\;P_{\mathrm{pkt}}=0.01\;W$$

Savings are quantified as:

$$PS\left(\%\right)=\frac{P_{\det}-P_{\mathrm{track}}}{P_{\det}}\times 100,\;BR\left(\%\right)=\frac{N_{\det}-N_{\mathrm{track}}}{N_{\det}}\times 100$$

#### 7.5.2. Results and Statistical Analysis

Table 16 presents a comprehensive comparison between detection-only and detection-with-tracking approaches. Tracking reduces the number of transmissions to the number of unique animals, achieving more than 99% BR and PS across all events.

Paired *t*-test (p<0.001) confirms statistical significance. Narrow CIs (±0.06% to ±0.12%) indicate high reproducibility.

#### 7.5.3. Discussion

Tracking at the edge eliminates duplicate detections, improving counting accuracy while reducing communication overhead by more than 99%. The statistical characterization (24 events, 95% CI, paired *t*-test) confirms these savings are genuine and reproducible, substantially enhancing network sustainability.

### 7.6. Operational Autonomy and Sustainability Metrics

To assess the practical viability of the extreme-edge node in a solar-constrained environment, we compute the system autonomy ($D_{aut}$). This metric represents the number of days the node can operate on a fixed battery capacity without any solar energy harvesting, such as during extended periods of poor weather conditions.

### 7.7. Autonomy Calculation Model

For this analysis, the energy storage system consists of two 12 V, 20 Ah batteries connected in parallel, providing an increased capacity suitable for long-term deployment.

The total battery capacity is given by:

$$C_{batt}=2\times \left(12\times 20\right)=480\;\mathrm{Wh}$$

Converting to kilojoules:

$$C_{batt\_kJ}=480\times 3.6=1728\;\mathrm{kJ}$$

The system autonomy is defined as:

$$D_{aut}=\frac{C_{batt\_kJ}}{E_{total}}$$

where $E_{total}$ is the daily energy consumption.

### 7.8. Comparative Autonomy Results

Table 17 summarizes the operational lifetime of both configurations.

The results clearly indicate that the event-driven PIR configuration significantly extends operational lifetime even under higher energy storage capacity. The system autonomy increases from 8.73 h to 178.3 h, demonstrating approximately a 20-fold improvement in endurance.

From a sustainability perspective, this extended autonomy reduces maintenance frequency and battery cycling, thereby lowering operational overhead and environmental impact. The combination of energy-efficient sensing and high-capacity storage enables long-term autonomous deployment in remote extreme-edge environments.

## 8. Real-Time Statistical Monitoring and Stakeholder Visualization

In this section, we describe the real-time monitoring and visualization framework implemented using Grafana at the edge server. The proposed dashboard system provides comprehensive visualization of wildlife monitoring data, environmental sensing information, network performance metrics, and system-level energy statistics collected from the deployed extreme edge nodes.

### 8.1. Software Integration for Communication and Node Failure Management

**Figure 10:**
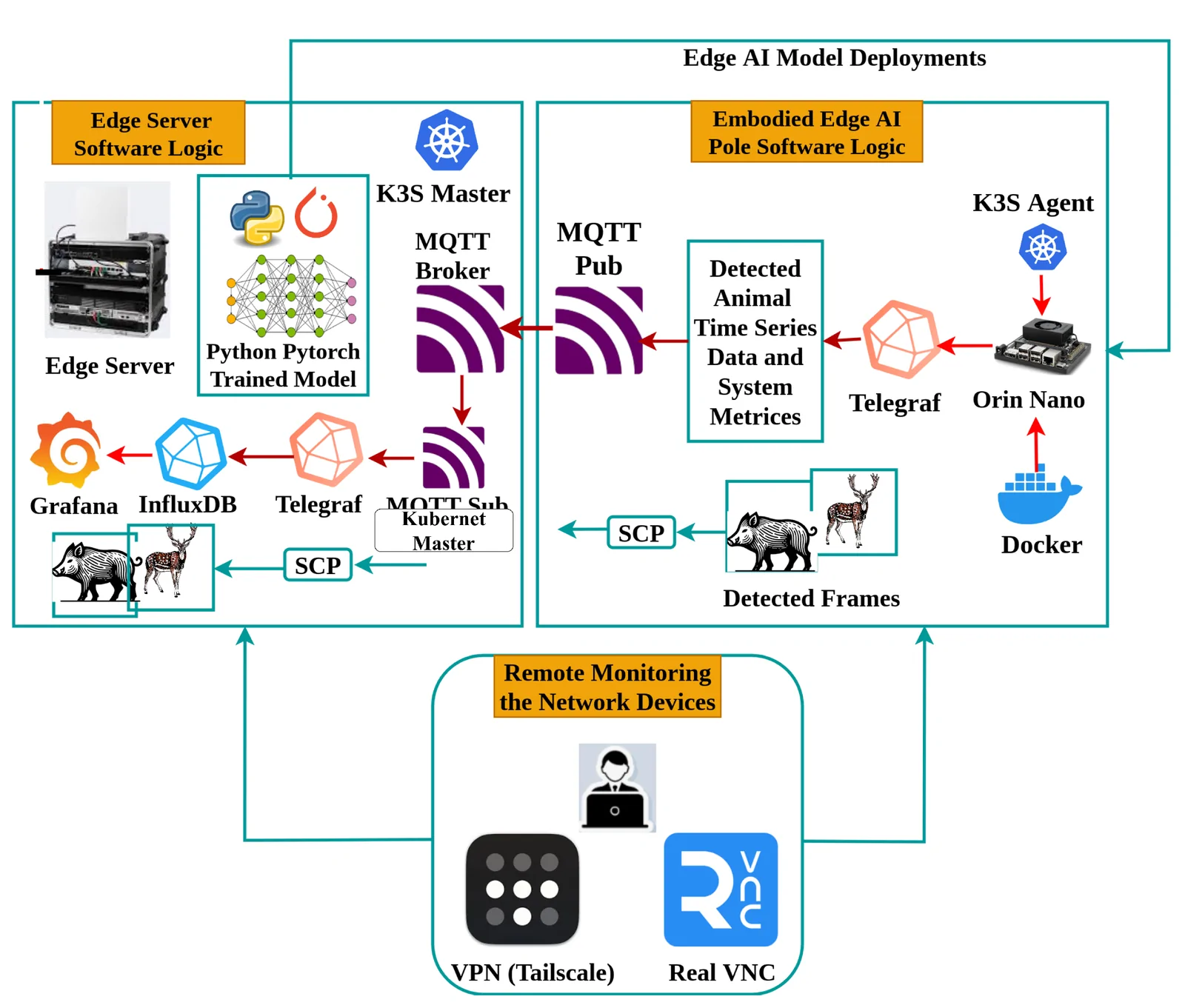
Software Integration for Collecting Data From Node to Edge Server.

In Figure 10, illustrates the communication software integration and fault-resilient operational framework of the proposed extreme-edge AI monitoring architecture. The system consists of an embedded edge AI pole node based on the NVIDIA Jetson Orin Nano and a centralized edge server operating within a K3S-based distributed management environment. The Orin Nano functions as a K3S agent and performs real-time AI inference inside Docker containers, while the edge server operates as the K3S master responsible for orchestration, monitoring, and communication management. The communication layer is primarily built upon the MQTT publish/subscribe protocol, enabling lightweight and scalable transmission of detected animal events, time-series telemetry, and system health information from the extreme-edge node to the edge server. Telegraf is deployed on both the edge node and the server to collect operational metrics such as CPU utilization, memory usage, network activity, and device status. These telemetry streams are forwarded to InfluxDB for persistent storage and visualized using Grafana dashboards to support real-time monitoring and performance analysis. In addition to telemetry communication, the architecture integrates secure copy protocol (SCP) for transferring detected image frames from the extreme-edge node to the centralized edge server. Instead of continuously transmitting image data, SCP-based transfer is scheduled during periods when the Jetson Orin Nano is not actively engaged in AI inference or event-processing tasks. This opportunistic transfer mechanism minimizes network congestion and reduces unnecessary communication overhead, thereby improving network sustainability and power efficiency in resource-constrained extreme-edge environments. By avoiding simultaneous high-load inference and bulk data transmission, the system maintains stable operational performance while extending the operating time of the edge node.

**Figure 11:**
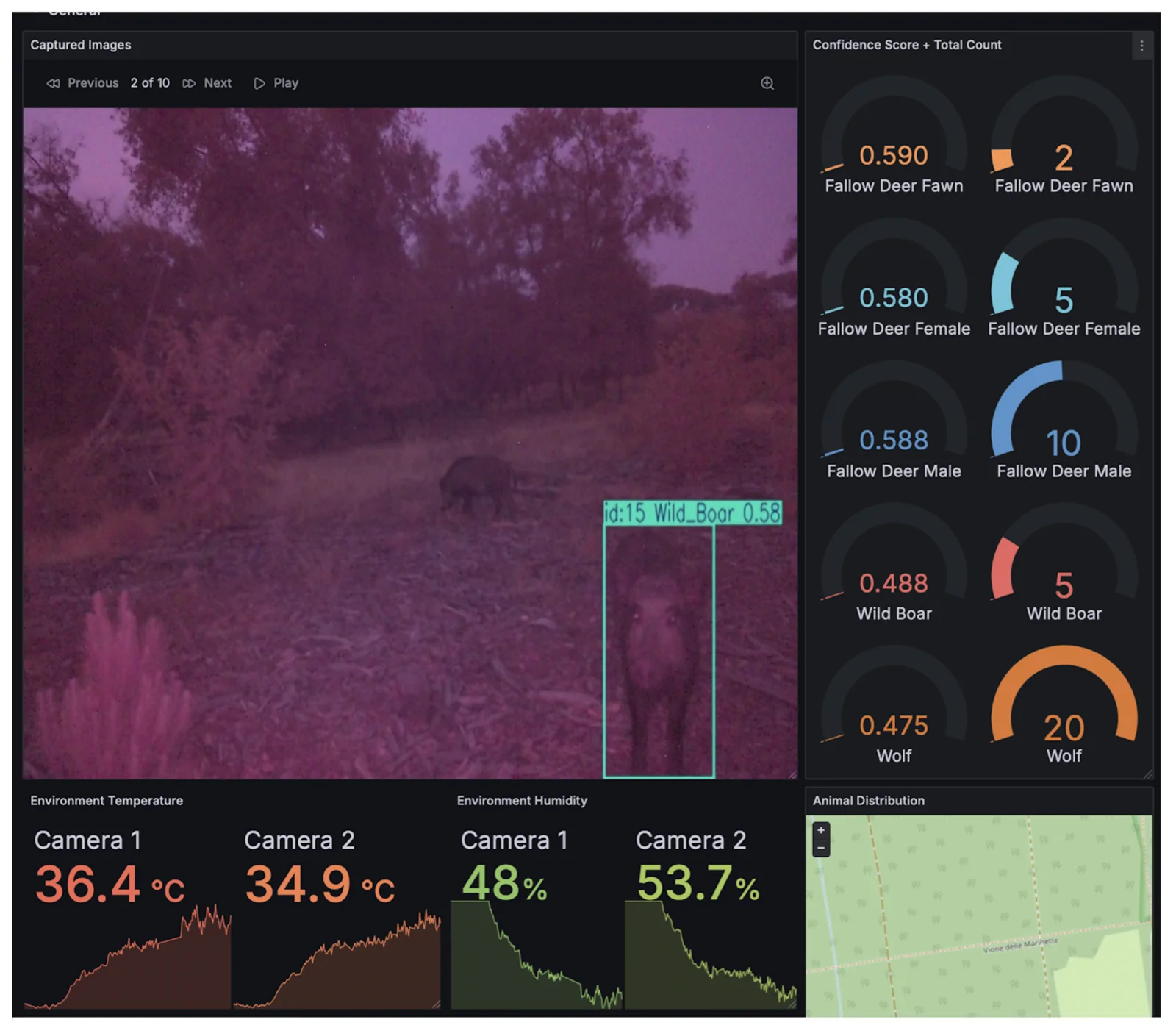
Grafana base Detected Animal visualization.

To ensure reliable remote accessibility and operational continuity in geographically isolated deployments, the architecture further incorporates VPN-based secure networking using Tailscale and remote maintenance through RealVNC. These components allow administrators to remotely diagnose failures, restart AI services, update Docker containers, and recover node functionality without physical access to the deployment site. The integration of K3S orchestration, MQTT messaging, SCP-based delayed image synchronization, telemetry monitoring, and remote management collectively provides a scalable, fault-aware, and energy-conscious communication infrastructure for long-term autonomous extreme-edge AI deployments. The Grafana-based visualization platform enables stakeholders to observe real-time wildlife events captured by the smart camera system, including detected animal classes, tracking IDs, confidence scores, and event timestamps, as shown in Figure 11. In addition, environmental monitoring parameters such as ambient temperature and humidity collected from onboard sensors are continuously streamed to the edge server for time-series analysis and displayed in the bottom-left corner of Figure 11.

**Figure 12:**
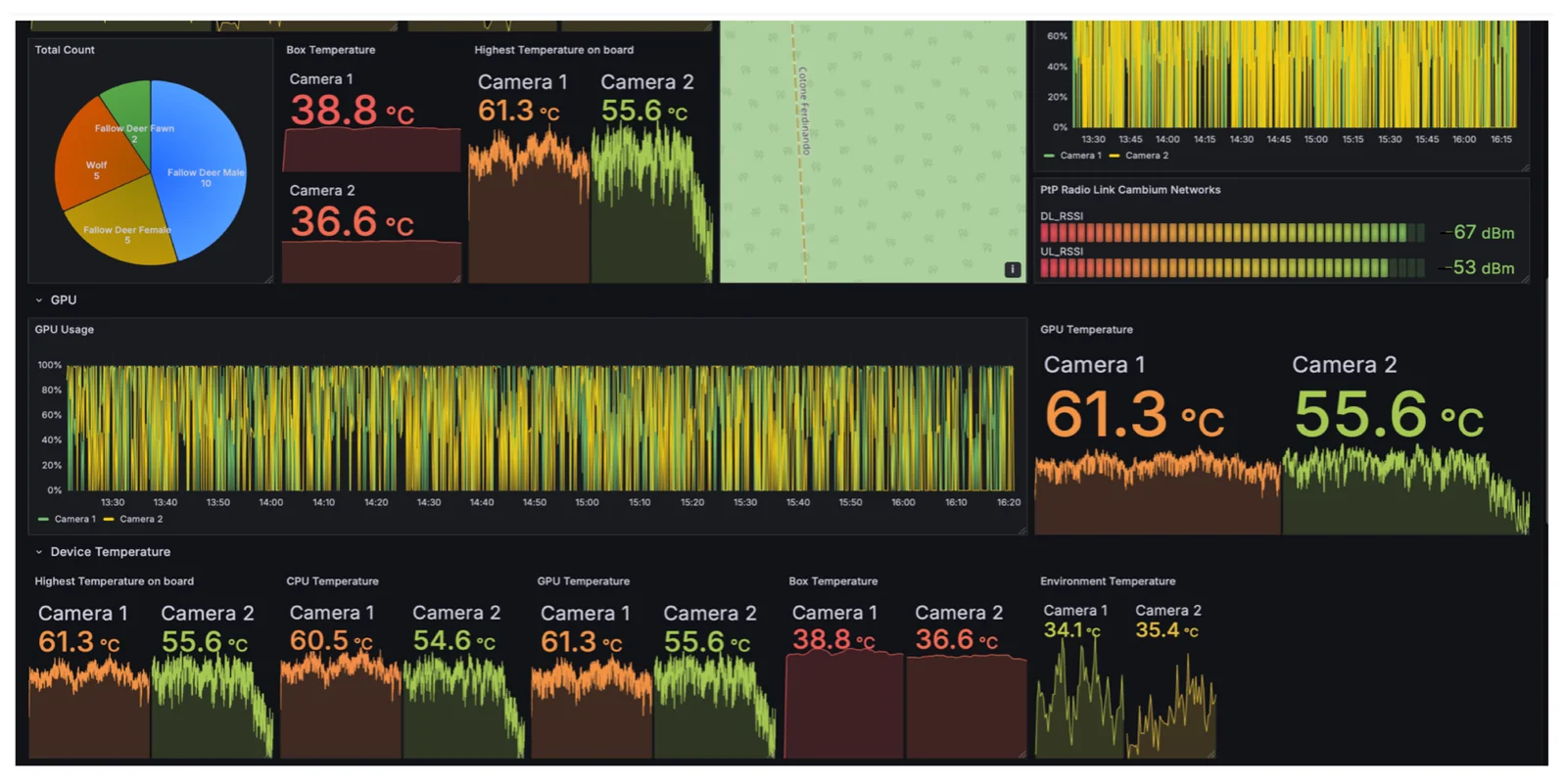
Processing Board Temperature (CPU, GPU, Box) Visualization.

Furthermore, in Figure 12 animal counts for selected wildlife events are displayed in the top-left corners of the images, while the bottom-left corners show system-level operational parameters, including Jetson board temperature, CPU and GPU utilization, and temperature records. The communication layer is also integrated into the visualization framework. Network metrics such as PTP link signal strength (dBm), uplink throughput, and downlink throughput are also included in this visualization.

**Figure 13:**
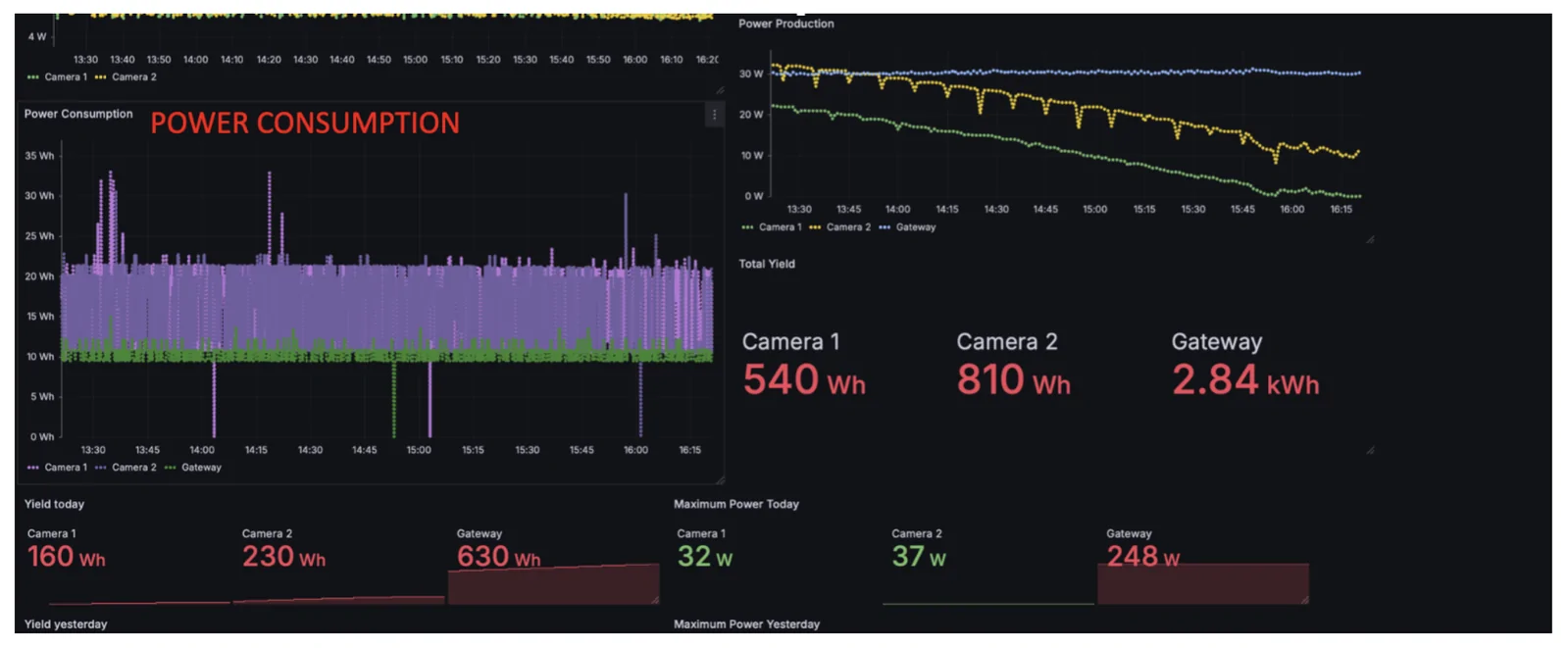
Solar Node Statistics Visualization.

In Figure 13, power consumption of each solar panel node, solar energy yield, battery charging status, and MPPT controller statistics are monitored to evaluate the energy efficiency and sustainability of the deployed extreme edge infrastructure.

By integrating wildlife analytics, environmental sensing, communication statistics, and energy management data into a unified visualization framework, the proposed system enables efficient monitoring by stakeholders, remote diagnostics, and long-term operational analysis. This real-time statistical monitoring framework therefore supports intelligent decision-making, predictive maintenance, and sustainability evaluation for autonomous extreme-edge AI deployments in remote wildlife monitoring environments.

### 8.2. Detection Challenges and Unseen-Class Observations at the Edge Server

**Figure 14:**
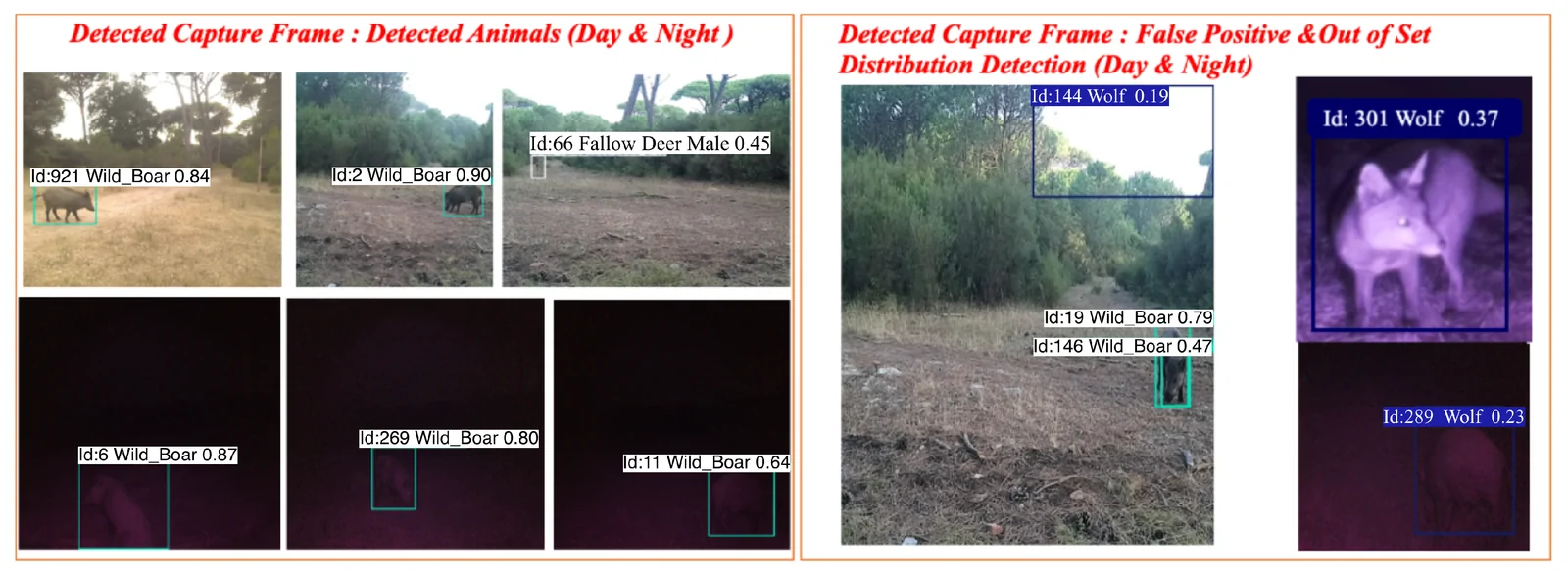
Detected animals captured from all deployed nodes and stored at the edge server.

In Figure 14, we present correctly classified and challenging detections captured under both daytime and nighttime conditions. A tree falsely detected as a wolf with 0.19 confidence represents a false positive driven by background complexity, in which structural patterns such as branches or shadows mimic animal silhouettes. A wild boar misclassified as a wolf with 0.23 confidence reflects closed-set confusion between known classes, suggesting that the discriminative features are insufficient to reliably distinguish morphologically similar species, particularly under low illumination.

Critically, a fox is classified as a wolf with 0.37 confidence at night—but since foxes were absent from the training set (limited to Fallow Deer Fawn, Female, Male, Wild Boar, and Wolf), this constitutes an open-set recognition failure, wherein the model maps an out-of-distribution (OOD) input to the nearest known class.

Notably, all these misclassifications exhibit very low confidence scores (0.19–0.37), significantly below the confidence levels of correct detections (typically >0.85). This suggests that a simple inference-time confidence threshold could effectively filter these errors. By rejecting detections with confidence scores below a predefined threshold (e.g., τ=0.5), the system could discard low-confidence predictions and flag them for human review rather than producing erroneous classifications. These results highlight the need for confidence-based filtering and out-of-distribution detection mechanisms in edge AI systems to ensure reliable wildlife monitoring under complex environmental conditions.

## 9. Conclusions and Future Work

This paper presented a sustainable AI-enabled extreme-edge wildlife monitoring system designed for long-term autonomous deployment in remote environments. The proposed architecture integrates event-driven sensing using a PIR sensor, quantized deep learning inference (FP32, FP16, and INT8), a split inference strategy for distributed processing across edge nodes, and a detection-with-tracking mechanism to minimize communication overhead. In addition, the system is powered by a solar-based energy harvesting and storage unit, enabling off-grid operation.

The experimental results demonstrate that PIR-based event-driven operation provides the largest contribution to energy efficiency, with an estimated 95.11% reduction in daily energy consumption compared with continuous operation, from 1320 Wh/day to 64.58 Wh/day, while maintaining functional responsiveness with only 150 s of active operation per day. Model quantization using INT8 precision delivers 4.7× higher throughput (29.8 FPS), 61% lower memory usage (1.2 GB), and 39% lower hardware power consumption (10.8 W) compared with FP32, with a modest 2.6% mAP reduction (92.3% to 89.7%). Field validation confirms the real-world effectiveness of the INT8 model with an overall mAP of 82.89% (86.40% daytime, 79.37% nighttime) and precision consistently above 96%.

The proposed tracking-based communication strategy achieves a reduction of more than 99% in packet transmissions and communication energy (p<0.001, paired *t*-test), with narrow confidence intervals (±0.06% to ±0.12%) confirming high reproducibility. Token-based channel allocation achieves 39.4% energy savings compared to TWT-based scheduling, with a sensitivity range of 29.2% to 45.1% depending on MAC-layer overhead conditions, validated through experimental measurements and sensitivity analysis. The split inference architecture enables distributed workload execution between edge devices, improving scalability and resource utilization, with the combined system achieving a minimum power consumption of 4.3 W when INT8 quantization and PIR activation are applied together.

The integration of renewable energy harvesting, energy-efficient sensing, lightweight AI inference, and communication-aware optimization enables scalable, autonomous, and sustainable wildlife monitoring in resource-constrained environments. The combined system achieves 63.76 Wh/day—a 95.17% reduction compared with FP32 continuous operation, with a 480 Wh battery providing 7.4 days of autonomy during overcast periods.

Future Work: Future research will focus on adaptive energy-aware inference scheduling, in which model precision (FP32, FP16, and INT8) is dynamically selected based on the available solar energy and battery state of charge. Adaptive quantization techniques that dynamically adjust precision based on available energy will also be explored to further optimize the energy–accuracy trade-off under varying environmental conditions. In addition, lightweight feature compression techniques will be investigated to further reduce communication overhead in the split inference architecture while preserving detection accuracy. The integration of on-device continual learning will also be explored to enable domain adaptation for new wildlife species and dynamically changing environmental conditions.

Furthermore, optimizing workload distribution and multi-node coordination strategies [45] for backbone–neck-head partitioning under varying network latency conditions remains an important research direction for improving scalability and robustness in large-scale deployments. Deploying the system across multiple nodes will be a key focus to validate scalability and coordination in large-scale wildlife monitoring networks. Secure authentication and authorization mechanisms will be investigated to strengthen communication security among distributed edge devices. In addition, federated learning [46] will be incorporated across multiple geographically distributed edge regions to enable collaborative model training while preserving data privacy and minimizing communication overhead.

Finally, the proposed framework will be extended to evaluate LEO satellite backhaul connectivity for remote wildlife monitoring [47], with emphasis on latency, bandwidth, reliability, energy consumption, and the impact of satellite communication on real-time distributed edge AI performance. Extending the dataset to include a wider range of species and environmental conditions will also be pursued to improve model generalization and robustness.

Overall, the proposed framework establishes a foundation for sustainable, intelligent, secure, and autonomous extreme-edge AI systems for long-term wildlife and environmental monitoring in resource-constrained and remote environments.

## Figures and Tables

**Table 1:** Comparison of Existing Works and Proposed Framework.

Reference	Methods	Dataset/ Application	Edge/Fog Architecture	Energy Efficient	Detection + Tracking	Quantization	Split Inference	Remote Management
[9]	Edge–Fog–Cloud Collaboration	IoT Systems	Yes	Yes	No	No	No	No
[11]	Hierarchical Edge–Fog–Cloud AI	AI-driven IoT	Yes	Partial	No	No	No	No
[3]	YOLO-based Edge AI Monitoring	Wildlife Monitoring	Yes	Partial	No	No	No	No
[13]	Energy-aware Node Selection	IoT Networks	Yes	Yes	No	No	No	No
[28]	INT8/FP16 Quantization	Edge AI Inference	No	Yes	No	Yes	No	No
[31]	Distributed Split Inference	Edge AI Systems	Yes	Partial	No	No	Yes	No
Proposed Work	Hierarchical Sustainable Network for AI	Wildlife Monitoring in Hostile Environments	Yes	Yes	Yes	Yes	Yes	Yes

**Table 2:** Comparison of Wireless Communication Technologies.

Technology	Typical Coverage/Distance	Bandwidth/Channel	Line of Sight	Typical Data Rate	Notes	Suitability
Wi-Fi 6 (802.11ax)	30–50 m indoors, 100 m outdoors	Up to 160 MHz per channel	LOS preferred	Up to 1.2 Gbps	Low latency, widely supported	Good for short-range edge deployments
Wi-Fi 6E/Wi-Fi 7	30–50 m indoors, 100 m outdoors	6 GHz band, wider channels	LOS preferred	Multi-Gbps	Ultra-low latency, emerging standard	Best for ultra-low latency, short range
Wi-Fi 5 (802.11ac)	30 m indoors, 100 m outdoors	Up to 160 MHz per channel	LOS preferred	Up to 1.3 Gbps	Older but common	Acceptable if devices only support Wi-Fi 5
Wi-Fi HaLow (802.11ah)	Up to 1 km outdoors, 100–200 m indoors	1–16 MHz, sub-GHz (900 MHz)	LOS improves range	Up to 347 Mbps ^1^ (theoretical PHY maximum under ideal conditions with 8 MHz channel and MIMO). For our narrow-channel, low-duty-cycle telemetry deployment, realistic throughput is significantly lower and range-dependent.		Suitable for 300 m wildlife park deployments with modest bandwidth requirements
5G NR (Standalone)	Several hundred meters	Variable (up to hundreds of MHz)	Not strictly required	1–10 Gbps (peak)	Needs 5G infra, low latency	Good if 5G infra is available
Wi-Fi Mesh	Large outdoor areas/campus	Depends on underlying Wi-Fi (6/6E/7)	LOS recommended	Depends on mesh nodes	Extends coverage via multiple nodes	Best for wide-area multi-Jetson setups
LoRa/LoRaWAN	10–15 km rural, 2–5 km urban	125–500 kHz (narrow)	LOS improves range	0.3–50 kbps	Ultra-low power, long range, not for tensors	Only for control/event triggers, not tensors

^1^ Theoretical PHY maximum under ideal conditions with 8 MHz channel and MIMO. In this deployment, measured throughput is 3.0–3.5 Mbps within 100–160 m range using narrow-channel, low-duty-cycle telemetry traffic. Actual throughput is range-dependent and significantly lower than the theoretical maximum.

**Table 3:** Power Consumption Breakdown by Component and Operation Mode.

Component	Power (W)	Operation	Continuous (W)	Event-Driven (W)
Sub-6 GHz Dish	15	24 h/day	15	0
Router/Switch	11	24 h/day	11	0
IEEE 802.11ah AP	4	Event-driven	0	4
Overhead	5	24 h/day	5	0
Total Peak	35	–	31	4

**Table 4:** Load Calculation of Extreme-Edge Node Components.

Component	Power (W)
Jetson Orin Nano (AI Processing)	15
Camera Module	4.5
IR LED Illumination	6
IEEE 802.11ah Transceiver	4
Sensors (DHT22 + Others)	2
PIR Sensor	0.4
MPPT Controller	3
DC Converter	3
System Losses (Cabling, conversion, margin)	5
Total (Active Mode)	42.9
Idle Mode (Optimized)	2.6

**Table 5:** Performance Comparison of YOLOv8m on Jetson Orin Nano.

Precision	FPS	GPU Load (%)	Power (W)	Memory (GB)
FP32	6.2	99	14.2	3.1
FP16	15.4	92	12.4	1.9
INT8	29.8	78	9.7	1.2

**Table 6:** Detection Accuracy Comparison Across Quantization Precisions (Lab Test Dataset).

Precision	mAP@0.5 (%)	Precision (%)	Recall (%)	Daytime mAP (%)	Nighttime mAP (%)
FP32	92.3	89.1	87.4	92.7	91.8
FP16	92.1	88.8	87.1	92.5	91.6
INT8	89.7	86.3	84.9	90.9	88.4

**Table 7:** Field Deployment Detection Accuracy of INT8 Model.

Video Type	Video (Duration/Content)	Frames	INT8 Deployed Model Detection Statistics Compared to Trail Camera GT						
			GT	TP	FP	FN	Precision (%)	Recall (%)	AP = mAP (%)
	7 s Fallow Deer (Fawn, Female)	245	980	910	18	70	98.06	92.86	92.86
Day Vision	8.5 s Fallow Deer Male	297	595	548	9	47	98.38	92.10	92.10
	6 s Wild Boar	210	420	308	25	112	92.49	73.33	73.33
Day Vision Average							96.31	86.40	86.40
	6.5 s Wild Boar	192	384	201	16	183	92.63	52.35	52.35
Night Vision	8 s Wild Boar	240	240	224	3	16	98.68	93.33	93.33
	7 s Wild Boar	210	420	382	7	38	98.20	90.95	90.95
Night Vision Average							96.50	79.37	79.37

**Table 8:** Projected Field mAP@0.5 (%) for FP32 Model.

Scenario	FP32 Field mAP (%)
Daytime Field mAP (%)	89.00
Nighttime Field mAP (%)	81.97
Overall Field mAP (%)	85.49

**Table 9:** System-Level Power and Energy Analysis for INT8 Deployed System.

Precision	$P_{\mathrm{SW}}$ (W) (Tegrastats)	$P_{\mathrm{HW}}$ (W) (MPPT)	Efficiency (%)	Without PIR (Wh/Day)	With PIR (Wh/Day)
FP32	14.2	17.8	79.7	1320	63.98
INT8 (Deployed)	9.7	10.8	89.8	801.24	63.76

**Table 10:** Derivation of Without PIR Daily Energy Values for INT8 Deployed System.

Precision	$P_{\mathrm{HW}}$ (W) (MPPT Measured)	Scaling Factor ($P_{\mathrm{HW}}$/17.8)	Calculation	Without PIR (Wh/Day)
FP32	17.8	1.000	55×24×1.000	1320
INT8	10.8	0.607	55×24×0.607	801.24

**Table 11:** Calculated Daily Energy Consumption under Continuous and Event-Driven Operation Based on Field-Observed Activity.

Scenario	Active Time (s)	Idle Time (s)	Active Power (W)	Idle Power (W)	Total Energy (J)	Energy (kJ)
Without PIR (Continuous)	86,400	0	55	0	4,752,000	4752.0
With PIR (Event-Driven)	150	86,250	55	2.6	232,500	232.5

**Table 12:** Performance and Power Comparison of Full vs. Split YOLOv8 Inference Architecture.

Configuration	Device Setup	Inference Power (W)	Comm. Power (W)	Total Power (W)	Observation
Full Model (Single Jetson)	1 Jetson Orin Nano	14.8	0	14.8	Baseline centralized inference
Split Model (Backbone + Neck-Head)	2 Jetsons (Distributed)	8.6 + 5.1	0.8	14.5	Workload distributed across nodes
Split Model + PIR (Event-Driven)	2 Jetsons + duty cycling	3.2 + 1.9	0.4	5.5	Significant reduction due to duty cycling
Split + PIR + Quantization (INT8)	2 Jetsons optimized	2.5 + 1.5	0.3	4.3	Maximum energy-efficient configuration

**Table 13:** Event-wise Packet Generation and Energy Comparison (Mean ± 95% CI).

Event Type	Frames	Detections/Frame	Packets	Proposed (J)	Baseline (J)
Wild Boar (Night) (*N* = 5)	192	2	384 ± 14	3.84 ± 0.14	5.38 ± 0.20
Wolf (Day) (*N* = 5)	502	2	1004 ± 26	10.04 ± 0.26	14.06 ± 0.36
Wolf (Night) (*N* = 5)	798	1	798 ± 18	7.98 ± 0.18	11.17 ± 0.25
Fallow Deer (Day) (*N* = 5)	522	5	2610 ± 52	26.10 ± 0.52	36.54 ± 0.73
Fallow Deer Fawn (Day) (*N* = 4)	380	3	1140 ± 30	11.40 ± 0.30	15.96 ± 0.42
Aggregated (*N* = 24)	–	–	–	11.53 ± 0.30	16.14 ± 0.42

**Table 14:** Sensitivity Analysis: Energy Savings vs. α.

α	Baseline Energy (J)	Savings (%)
1.2	14.39	29.2
1.3	15.59	34.7
1.4	16.79	39.4
1.5	17.99	43.4
1.6	19.18	45.1

**Table 15:** Measured TWT Overhead Factor α.

Traffic Regime	Events/Hour	Measured α (Mean ± SD)
Low	1–2	1.12 ± 0.05
Medium	5–10	1.28 ± 0.08
High	15–20	1.42 ± 0.10

**Table 16:** Detection-Only vs. Detection-with-Tracking (Mean ± 95% CI).

Event	Frames	Detections	Tracks	Power (W)	Track Power (W)	Saved Power (W)	BR (%)	PS (%)
Wild Boar (Night) (*N* = 5)	192	384 ± 14	2	3.84 ± 0.14	0.02 ± 0.001	3.82 ± 0.14	99.48 ± 0.12	99.48 ± 0.12
Wolf (Day) (*N* = 5)	502	1004 ± 26	2	10.04 ± 0.26	0.02 ± 0.001	10.02 ± 0.26	99.80 ± 0.08	99.80 ± 0.08
Wolf (Night) (*N* = 5)	798	798 ± 18	1	7.98 ± 0.18	0.01 ± 0.001	7.97 ± 0.18	99.87 ± 0.06	99.87 ± 0.06
Fallow Deer (Day) (*N* = 5)	522	2610 ± 52	5	26.10 ± 0.52	0.05 ± 0.002	26.05 ± 0.52	99.81 ± 0.07	99.81 ± 0.07
Fallow Deer Fawn (Day) (*N* = 4)	380	1140 ± 30	3	11.40 ± 0.30	0.03 ± 0.001	11.37 ± 0.30	99.74 ± 0.09	99.74 ± 0.09

**Table 17:** System Autonomy Comparison (Two 12 V, 20 Ah Batteries).

Scenario	$E_{\mathrm{total}}$ (kJ/Day)	$D_{\mathrm{aut}}$ (Days)	Runtime (Hours)
Without PIR (Continuous)	4752	0.36	8.73
With PIR (Event-Driven)	232.5	7.43	178.3

## Data Availability

The data presented in this study are available on request from the first author. The data are not publicly available due to privacy restrictions.

## References

[1] Caro T. (1998). Behavioral Ecology and Conservation Biology.

[2] Kays R., Crofoot M.C., Jetz W., Wikelski M. (2015). Terrestrial animal tracking as an eye on life and planet. Science.

[3] Rahman M.A., Mehari Berhe T., Borgianni L., Sabbir Ahmed M., Bua C., Adami D., Giordano S. (2025). A Scalable Framework for Deploying AI-Powered Wildlife Monitoring in Resource-Limited Field Environments. IEEE Access.

[4] Olawade D.B., Wada O.Z., Ige A.O., Egbewole B.I., Olojo A., Oladapo B.I. (2024). Artificial intelligence in environmental monitoring: Advancements, challenges, and future directions. Hyg. Environ. Health Adv..

[5] Redmon J., Divvala S., Girshick R., Farhadi A. (2016). You Only Look Once: Unified, Real-Time Object Detection. Proceedings of the 2016 IEEE Conference on Computer Vision and Pattern Recognition (CVPR).

[6] Shi W., Dustdar S. (2016). The Promise of Edge Computing. Computer.

[7] Mao Y., You C., Zhang J., Huang K., Letaief K.B. (2017). A Survey on Mobile Edge Computing: The Communication Perspective. IEEE Commun. Surv. Tutor..

[8] Satyanarayanan M. (2017). The Emergence of Edge Computing. Computer.

[9] Fernando N., Shrestha S., Loke S.W., Lee K. (2025). On Edge-Fog-Cloud Collaboration and Reaping Its Benefits: A Heterogeneous Multi-Tier Edge Computing Architecture. Future Internet.

[10] Dautov R., Distefano S. (2017). Three-level hierarchical data fusion through the IoT, edge, and cloud computing. Proceedings of the 1st International Conference on Internet of Things and Machine Learning.

[11] Firouzi F., Farahani B., Marinšek A. (2022). The convergence and interplay of edge, fog, and cloud in the AI-driven Internet of Things (IoT). Inf. Syst..

[12] Babar M., Khan M.S. (2021). ScalEdge: A framework for scalable edge computing in Internet of things–based smart systems. Int. J. Distrib. Sens. Netw..

[13] Fereira R., Ranaweera C., Lee K., Schneider J.G. (2023). Energy Efficient Node Selection in Edge-Fog-Cloud Layered IoT Architecture. Sensors.

[14] Cao J. (2026). Intelligent computing architectures for sustainable large-scale IoT ecosystems enabling AI-driven industrial transformation. Discov. Internet Things.

[15] Meera M., Rao S.N. (2017). A Survey of the State of the Art of 802.11ah. Proceedings of the 2017 IEEE International Conference on Computational Intelligence and Computing Research (ICCIC).

[16] Wang H., Fapojuwo A.O. (2017). A Survey of Enabling Technologies of Low Power and Long Range Machine-to-Machine Communications. IEEE Commun. Surv. Tutor..

[17] Adame T., Bel A., Bellalta B., Barcelo J., Oliver M. (2014). IEEE 802.11ah: The Wi-Fi Approach for M2M Communications. IEEE Wirel. Commun..

[18] Santi S., Tian L., Khorov E., Famaey J. (2019). Accurate Energy Modeling and Characterization of IEEE 802.11ah RAW and TWT. Sensors.

[19] Ahmed N., De D., Barbhuiya F.A., Hussain M.I. (2021). MAC Protocols for IEEE 802.11ah-Based Internet of Things: A Survey. IEEE Internet Things J..

[20] Chang T.C., Lin C.H., Lin C.J., Chen W.T. (2019). Traffic-Aware Sensor Grouping for IEEE 802.11ah Networks: Regression Based Analysis and Design. IEEE Trans. Mob. Comput..

[21] Mahesh M., Harigovindan V. (2019). Fuzzy-Based Optimal and Traffic-Aware Restricted Access Window Mechanism for Dense IoT Networks. J. Intell. Fuzzy Syst..

[22] Handley M. (2018). Delay is Not an Option: Low Latency Routing in Space. Proceedings of the 17th ACM Workshop on Hot Topics in Networks.

[23] Santos C., Jiménez J.A., Espinosa F. (2019). Effect of Event-Based Sensing on IoT Node Power Efficiency. Case Study: Air Quality Monitoring in Smart Cities. IEEE Access.

[24] Kong L., Tan J., Huang J., Chen G., Wang S., Jin X., Zeng P., Khan M., Das S.K. (2022). Edge-computing-driven Internet of Things: A Survey. ACM Comput. Surv..

[25] NVIDIA Corporation (2023). Jetson Orin Nano Developer Kit User Guide.

[26] Wojke N., Bewley A., Paulus D. (2017). Simple online and realtime tracking with a deep association metric. Proceedings of the 2017 IEEE International Conference on Image Processing (ICIP).

[27] (2022). Multi-object tracking in traffic environments: A systematic literature review. Neurocomputing.

[28] Sahu I., Pal A., Ukil A., Majumdar A. (2021). Compressing Deep Neural Network: A Black-Box System Identification Approach. Proceedings of the 2021 International Joint Conference on Neural Networks (IJCNN).

[29] Micikevicius P., Narang S., Alben J., Diamos G.F., Elsen E., García D., Ginsburg B., Houston M., Kuchaiev O., Venkatesh G. (2017). Mixed Precision Training. arXiv.

[30] Kuzmin A., Nagel M., van Baalen M., Behboodi A., Blankevoort T. (2023). Pruning vs. quantization: Which is better?. Proceedings of the 37th International Conference on Neural Information Processing Systems.

[31] Rahman M.A., Adami D., Giordano S. (2025). Deep Learning Model Splitting for Distributed Inference in Resource-Constrained Edge Environments for Wildlife Monitoring. Proceedings of the 2025 28th International Conference on Computer and Information Technology (ICCIT).

[32] Merlino G., Dautov R., Distefano S., Bruneo D. (2019). Enabling Workload Engineering in Edge, Fog, and Cloud Computing through OpenStack-based Middleware. ACM Trans. Internet Technol..

[33] Urblik L., Kajati E., Papcun P., Zolotová I. (2024). Containerization in Edge Intelligence: A Review. Electronics.

[34] Pahl C., Lee B. (2015). Containers and Clusters for Edge Cloud Architectures—A Technology Review. Proceedings of the 2015 3rd International Conference on Future Internet of Things and Cloud.

[35] Morabito R. (2018). Evaluating Performance of Containerized IoT Services for Clustered Devices at the Network Edge. IEEE Internet Things J..

[36] Medel V., Tolosana-Calasanz R., Bañares J.Á., Arronategui U., Rana O.F. (2018). Characterising Resource Management Performance in Kubernetes. Comput. Electr. Eng..

[37] Skarlat O., Nardelli V., Schulte S., Dustdar S. (2017). Towards QoS-Aware Fog Service Placement. Proceedings of the 2017 IEEE 1st International Conference on Fog and Edge Computing (ICFEC).

[38] Rahman M.A., Giordano S., Pagano M. (2025). Smart Wildlife Monitoring: Real-Time Hybrid Tracking Using Kalman Filter and Local Binary Similarity Matching on Edge Network. Computers.

[39] Rahman M.A., Giordano S., Pagano M. (2026). Smart Wildlife Monitoring: Power and Bandwidth Efficient Real-Time Object Tracking with Local Binary Similarity Matching on Resource Constrained Edge Network. Proceedings of the Applications in Electronics Pervading Industry, Environment and Society.

[40] Rahman M.A., Berhe T.M., Giordano S. (2026). European Animal Detection Dataset. https://ieee-dataport.org/documents/european-animal-detection-dataset.

[41] Masters G.M. (2013). Renewable and Efficient Electric Power Systems.

[42] Duffie J.A., Beckman W.A. (2013). Solar Engineering of Thermal Processes.

[43] Skoplaki E., Palyvos J. (2009). On the temperature dependence of photovoltaic module electrical performance: A review of efficiency/power correlations. Sol. Energy.

[44] Khorov E., Lyakhov A., Krotov A., Guschin A. (2015). A survey on IEEE 802.11ah: An enabling networking technology for smart cities. Comput. Commun..

[45] Baneriee A., Chishti M.S., Rahman A., Chanazain R. (2018). Centralized Framework for Workload Distribution in Fog Computing. Proceedings of the 2018 3rd International Conference for Convergence in Technology (I2CT).

[46] Rahman M.A., Debnath A.C., Giordano S. (2024). Model Poisoning Detection and Dismissal Safeguarding Federated Learning Against Malicious Attacks. Proceedings of the 2024 IEEE 29th International Workshop on Computer Aided Modeling and Design of Communication Links and Networks (CAMAD).

[47] Rahman M.A., Borgianni L., Marku E., Mathew S., Navadehrazi O., Coli E., Adami D., Giordano S. (2025). Monitoring Wildlife in Remote Areas through LEO Satellites within a Cloud Continuum Perspective. Proceedings of the 2025 IEEE Globecom Workshops (GC Wkshps).

